# Quartet: Disentangling positive and negative components of microbial interactions

**DOI:** 10.1371/journal.pcbi.1014502

**Published:** 2026-07-10

**Authors:** Aamir Faisal Ansari, Gayathri Sambamoorthy, Thrisha C. Alexander, Yugandhar B. S. Reddy, Janhavi Raut, Narendra M. Dixit

**Affiliations:** 1 Department of Chemical Engineering, Indian Institute of Science, Bengaluru, Karnataka, India; 2 Unilever R&D India Pvt Ltd, Bengaluru, Karnataka, India; 3 Department of Bioengineering, Indian Institute of Science, Bengaluru, Karnataka, India; University of Nebraska-Lincoln, UNITED STATES OF AMERICA

## Abstract

Interspecies interactions are characterized conventionally by the net influence, positive or negative, a species exerts on another. Community ecology theories rely on these net interactions to describe the behaviour of multispecies communities. The net interactions in turn comprise positive and negative components, arising typically from cross-feeding metabolites and competition for resources. The components remain challenging to disentangle, compromising descriptions of community behaviour. Here, we devised a method to estimate the components when metabolic interactions predominate. We conceived a theoretical resource partitioning strategy which when applied to data on species growth rates disentangles the components. Consequently, the net influence a species has on another is decomposed into its positive and negative components. The interactions between a pair of species are thus defined by the ‘quartet’ of underlying components, specifically the positive and negative components of the net influence of each species on the other. We applied the method to 28 *in silico* species pairs from a representative oral microbiome and an experimental auxoptroph pair from the literature. We found that positive and negative components had comparable strengths on average. Interestingly, we found species pairs with similar net interactions but disparate components, highlighting the importance of the quartet. Further, weak net interactions could arise from cancellation of strong components. Estimating the quartet helped better understand the complex transitions in community behaviour observed upon varying resource supply *in silico* and *in vitro*. The quartet thus offers a more fundamental characterization of interspecies interactions and may help build more reliable community ecology theories, with implications for understanding and design of microbial communities.

## Author summary

Multispecies microbial communities are important to healthcare, the environment, and biotechnology. Their behaviour depends on the interactions between the species involved. The interactions have been characterized conventionally by the net influence, positive or negative, a species has on another. This conventional view has limitations because the net influence is often made up of positive and negative underlying components, arising, for instance, from cross-feeding metabolites and competition for resources, respectively. Disentangling the components has been challenging. Here, we developed a method for disentangling the components when metabolic interactions predominate. The net interactions between species pairs are then replaced by the more fundamental ‘quartet’ of their components. The quartet explains several confounding experimental observations, facilitating better understanding and engineering of microbial communities.

## Introduction

Multi-species microbial communities are important to human health [1], environmental sustainability [2,3], and biotechnology [4,5]. Establishing and maintaining stable microbial communities relies on the knowledge of the interactions between the constituent species [6,7]. The interactions have proven challenging to unravel and understand [7–13]. Conventionally, the interactions between a pair of species have been categorized by the nature, positive or negative, of the effect each species has on the survival and/or growth of the other. When the effects are both positive (+,+), *i.e.*, when the species benefit each other, the species are said to exhibit cooperation, or mutualism. When the effects are both negative (-,-), they exhibit competition. Other combinations are termed exploitation or parasitism (+,-), commensalism (+,0), amensalism (-,0), and neutralism (0,0), where ‘0’ implies no effect. Current community ecology theories, beginning with the seminal work of May [14], predict the behaviour of communities, including their stability, based on this conventional characterization [7,9,15,16]. For instance, competitive interactions, particularly exploitative ones like the predator-prey interactions, are predicted to be stabilizing as they introduce negative feedbacks, whereas cooperative interactions may introduce dependencies that may render communities unstable [7,9,15].

This conventional characterization has a fundamental limitation: It adopts an ‘either-or’ view of positive and negative interactions. A species, in contrast, can simultaneously exert both positive and negative influences on another. In many ecological niches and synthetic communities, for instance, interactions are predominantly metabolic in nature [17–22]. In such scenarios, negative interactions typically arise from competition for resources and positive interactions from cross-feeding metabolites. Both these interactions are frequently known to occur together [17,21–23]. Positive and negative interactions are thus not exclusive. The net interactions observed experimentally are the resultants of these underlying competing influences. Indeed, the net interactions between the same species pairs can be either positive or negative depending on environmental conditions [11,17,21,22,24], reiterating the ability of species pairs to engage in both positive and negative interactions simultaneously. Hence, employing the net interactions to characterize communities while ignoring the underlying components can limit our understanding of communities, compromise the predictive power of theories, and preclude robust community design.

Overcoming the limitation of the conventional characterization requires ways of estimating the underlying positive and negative components. While net interactions are readily measured for culturable species [8,10,21,22,24], methods to disentangle the components are only beginning to be developed. Genome-scale metabolic models and other computational approaches have often deduced the potential for positive and negative interactions [17,23,25,26]. For instance, the fraction of exogenous metabolites shared between a species pair was used to define a competition index, whereas the fraction of metabolites used by one species but produced by the other in the pair yielded a complementarity index [26]. Such empirical measures offer insights into the components but do not quantify them. More recently, a method, termed kinetics-based inference of dynamic variation in microbial interactions (KIDI), has been developed to estimate the components when cross-feeding metabolites and shared limiting nutrients are known along with their kinetic relationships with species growth rates [27]. This development marks an important advance. Often, however, knowledge of the metabolites involved and their kinetic relationships is not available, limiting the scope of KIDI.

Here, we developed a broadly applicable method to estimate the positive and negative components of the interactions between a species pair. It is conceptually distinct from existing approaches. The method employs data on species growth rates, which are more readily obtained, either by measurement or by estimation using computational methods, than the data required by extant methods. It then constructs a theoretical partitioning of resources which enables estimation of the positive components. The negative components follow from knowledge of the net interactions. Thus, each net interaction is decomposed into its positive and negative components. Conventionally, as discussed above, a species pair has been characterized by the pair of their net interactions, yielding the broad categorizations of mutualism, competition, and so on. Here, with the two components of each of the net interactions identified, the species pair is characterized by the ‘quartet’ of the components: the positive and negative components of the net interaction of each species with the other. We demonstrate the utility of the method by applying it to an *in silico* representative oral microbiome and a published experimental dataset on a 2-species community of auxotrophs.

## Results

### Method to estimate the positive and negative components of species interactions

**Fig 1:**
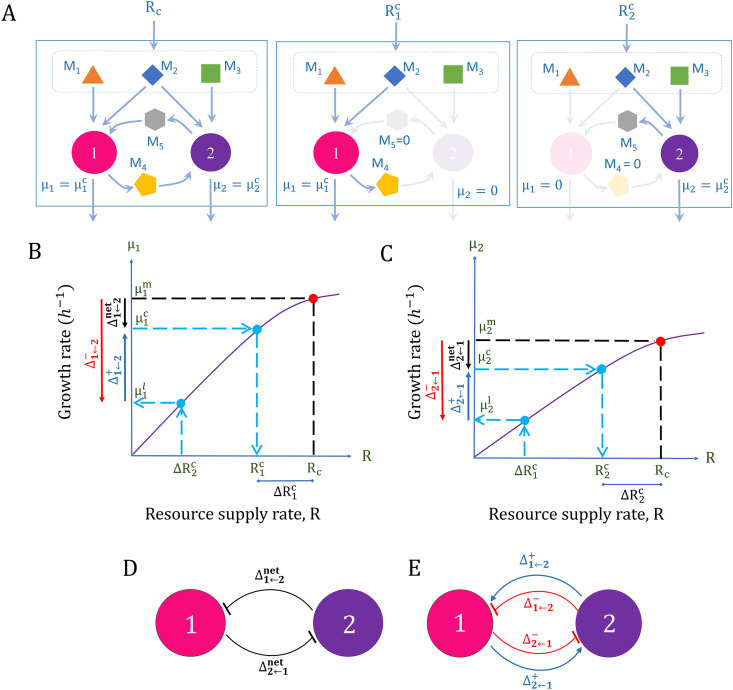
Method to estimate the positive and negative components of the net interactions between species. (A) A hypothetical two-species community supplied with nutrients, M_1_, M_2_, and M_3_, at constant rates, with $R_{c}$ the supply rate of the shared limiting resource, M_2_ (left). Metabolite M_4_, produced by species 1 (pink) and consumed by species 2 (purple), and metabolite M_5_, with the opposite pattern, are cross-feeding metabolites. The species growth rates in the community are $\mu_{\text{1}}^{c}$ and $\mu_{\text{2}}^{c}$, respectively. The resource supply rates that support the species individually at their community growth rates are $R_{\text{1}}^{c}$ (middle) and $R_{\text{2}}^{c}$ (right). (B & C) The growth-resource curves of species 1 (B) and species 2 (C) in monoculture. $\mu_{\text{1}}^{m}$ and $\mu_{\text{2}}^{m}$ are the monoculture growth rates of the species at the resource supply rate $R_{c}$. The leftover resources, $\Delta R_{\text{1}}^{c}$ and $\Delta R_{\text{2}}^{c}$, yield the associated growth rates $\mu_{\text{1}}^{l}$ and $\mu_{\text{2}}^{l}$. The interaction components estimated by our method (see text) are illustrated next to the y-axis. (D) A depiction of the net interactions employed conventionally. (E) A depiction of the quartet of components estimated here.


We consider a two-species community, comprising species ‘1’ and ‘2’, in an environment supplied with nutrients (or resources) at a constant rate, $R_{c}$ (Fig 1A). The resources may contain ingredients that are exclusive to the individual species (e.g., M_1_ and M_3_ in Fig 1A) or shared by both (e.g., M_2_ in Fig 1A). Further, the species may also exchange, or cross-feed, metabolites they produce (e.g., M_4_ and M_5_ in Fig 1A). As a result of all these interactions, the species may grow. We denote the steady-state growth rates of the species in the community environment as $\mu_{\text{1}}^{c}$ and $\mu_{\text{2}}^{c}$, respectively, where ‘c’ refers to the community. In the same environment, we denote the steady-state growth rates of the species when present alone, or in monoculture, as $\mu_{\text{1}}^{m}$ and $\mu_{\text{2}}^{m}$, respectively. If the species experienced no interactions, the monoculture and community growth rates of the respective species would be identical. A difference between the two rates indicates the presence of net interactions between the species. Following convention [7], we define the net influence of species ‘2’ on species ‘1’ as:

$$\begin{array}{c} \begin{array}{c} \Delta_{\text{1}\leftarrow \text{2}}^{net}=\mu_{\text{1}}^{c}-\mu_{\text{1}}^{m} \end{array} \end{array}$$ (1)

$\Delta_{\text{1}\leftarrow \text{2}}^{net}>\text{0}$ implies an overall positive effect of species 2 on species 1, as the growth rate of species 1 in the community is greater than in monoculture. Accordingly, $\Delta_{\text{1}\leftarrow \text{2}}^{net}<\text{0}$ implies a net negative effect. Similarly, we define the net influence of species ‘1’ on species ‘2’ as


$$\begin{array}{c} \begin{array}{c} \Delta_{\text{2}\leftarrow \text{1}}^{net}=\mu_{\text{2}}^{c}-\mu_{\text{2}}^{m} \end{array} \end{array}$$ (2)

This pair of net interactions ($\Delta_{\text{1}\leftarrow \text{2}}^{net}$, $\Delta_{\text{2}\leftarrow \text{1}}^{net}$) yields the conventional characterization of interspecies interactions summarized above. Our goal is to estimate the positive and negative components of these net interactions.

The method we devised is based on the following conceptual arguments. We consider the scenario where negative interactions arise from competition for shared resources. How shared resources are partitioned between species is not typically known [17,22,28], leaving the negative components difficult to quantify. We conceived a theoretical partitioning that would prescribe bounds on the negative components and thereby offer estimates of the positive components. Accordingly, we let species 2 receive all the resources necessary for sustaining its growth at the rate observed in the community. The ‘left-over’ resources would then be available for use by species 1. Using knowledge of how the growth rate of species 1 depends on resource availability, we estimate its growth rate that the left-over resources can sustain. If the latter growth rate is smaller than the observed growth rate of species 1, then it would imply that species 1 is sustained additionally by cross-feeding metabolites from species 2 in the community. Indeed, the difference between the observed growth rate and the growth rate from the left-over resources would be an estimate of the positive component of the influence of species 2 on species 1. In the same way, with another theoretical partitioning where species 1 gets all the resources needed to sustain its growth in the community, one can estimate the positive component of the net influence species 1 has on species 2. The difference between the net influence and the positive component would yield the negative component. Below, we formalize these conceptual arguments into a procedure for estimating the components.

We denote the supply rate of the shared limiting resource in the environment as *R*. Species growth rates are expected to increase monotonically with *R*. We define the curve depicting the growth rate, μ, of a species in monoculture as a function of *R* as the ‘growth-resource curve’ of the species, denoted μ(R). For the species 1 and 2, we denote the growth-resource curves as $\mu_{\text{1}}(R)$ and $\mu_{\text{2}}(R)$, respectively. These curves are readily determined using experiments, or using genome-scale metabolic models where available, and are analogous to the classical description of the growth rate dependent on the substrate concentration due to Monod [29]. We recall that we consider an environment supplied with resources at a constant rate, $R_{c}$ (Fig 1A). At this resource supply rate, the respective monoculture growth rates of the two species would be $\mu_{\text{1}}^{m}=\mu_{\text{1}}(R_{c})$ and $\mu_{\text{2}}^{m}=\mu_{\text{2}}(R_{c})$ (Fig 1B and 1C).

Using the growth-resource curve of species 2, we estimate the resource supply rate, $R_{\text{2}}^{c}$, at which the growth rate of species 2 in monoculture would equal its growth rate in the community, $\mu_{\text{2}}^{c}$ (Fig 1C). In other words, we find $R_{\text{2}}^{c}$ as the value of *R* that satisfies $\mu_{\text{2}}(R)=\mu_{\text{2}}^{c}$. If $R_{\text{2}}^{c}<R_{c}$, it implies that not all of the resources supplied to the community were needed for the maintenance of species 2. The remaining, or left-over, resource supply rate, $R_{c}-R_{\text{2}}^{c}$, denoted $\Delta R_{\text{2}}^{c}$, would therefore be available to species 1 (Fig 1C). (Note that if $R_{\text{2}}^{c}\geq R_{c}$, then we set $\Delta R_{\text{2}}^{c}=\text{0}$; the left-over resource cannot be negative.) We now use the growth-resource curve of species 1 and identify its growth rate in monoculture afforded by the left-over resource supply rate $\Delta R_{\text{2}}^{c}$, which we denote as $\mu_{\text{1}}^{l}$ (Fig 1B). Thus, $\mu_{\text{1}}^{l}=\mu_{\text{1}}(\Delta R_{\text{2}}^{c})$. In other words, $\mu_{\text{1}}^{l}$ would be the growth rate of species 1 that the environment would sustain ‘after’ letting species 2 exist at its community growth rate in the absence of additional interactions between the two species. Clearly, the difference between $\mu_{\text{1}}^{l}$ and the observed growth rate, $\mu_{\text{1}}^{c}$, must be due to the positive effect of species 2 on species 1. We thus estimate the positive component of the interaction as


$$\begin{array}{c} \begin{array}{c} \Delta_{\text{1}\leftarrow \text{2}}^{+}=\mu_{\text{1}}^{c}-\mu_{\text{1}}^{l}\; \end{array} \end{array}$$ (3)

The difference between the net interaction, estimated above (Eq. 1), and the positive component yields the negative component:


$$\begin{array}{c} \begin{array}{c} \Delta_{\text{1}\leftarrow \text{2}}^{-}=\Delta_{\text{1}\leftarrow \text{2}}^{net}-\Delta_{\text{1}\leftarrow \text{2}}^{+}\; \end{array} \end{array}$$ (4)

In the same way, we estimate the components for the effect of species 1 on species 2: We employ the growth-resource curve of species 1 to obtain the resource supply rate, $R_{\text{1}}^{c}$, at which its monoculture growth rate would equal $\mu_{\text{1}}^{c}$ (Fig 1B); i.e., we identify $R_{\text{1}}^{c}$ as that value of *R* at which $\mu_{\text{1}}(R)=\mu_{\text{1}}^{c}$. The left-over resource, $\Delta R_{\text{1}}^{c}=R_{c}-R_{\text{1}}^{c}$, would be available to species 2 after letting species 1 grow at its community growth rate, $\mu_{\text{1}}^{c}$. (Again, note that $\Delta R_{\text{1}}^{c}\geq \text{0}$.) From the growth-resource curve of species 2 (Fig 1C), the growth rate, $\mu_{\text{2}}^{l}$, of species 2 at the left-over resource supply rate $\Delta R_{\text{1}}^{c}$ is estimated as $\mu_{\text{2}}^{l}=\mu_{\text{2}}(\Delta R_{\text{1}}^{c})$. The positive component of the net influence of species 1 on species 2 is then


$$\begin{array}{c} \begin{array}{c} \Delta_{\text{2}\leftarrow \text{1}}^{+}=\mu_{\text{2}}^{c}-\mu_{\text{2}}^{l} \end{array} \end{array}$$ (5)

and the negative component,


$$\begin{array}{c} \begin{array}{c} \Delta_{\text{2}\leftarrow \text{1}}^{-}=\Delta_{\text{2}\leftarrow \text{1}}^{net}-\Delta_{\text{2}\leftarrow \text{1}}^{+}\; \end{array} \end{array}$$ (6)

Together, the components $\Delta_{\text{1}\leftarrow \text{2}}^{+}$, $\Delta_{\text{1}\leftarrow \text{2}}^{-}$, $\Delta_{\text{2}\leftarrow \text{1}}^{+}$, and $\Delta_{\text{2}\leftarrow \text{1}}^{-}$ form the quartet of interactions between the two species in the community environment (Fig 1D and 1E). Below, we demonstrate the procedure by applying it to a specific species pair.

### The quartet of interactions between *Streptococcus mutans* and *Streptococcus parasanguinis*

**Fig 2:**
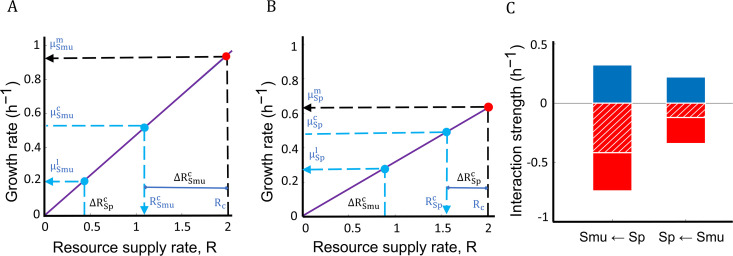
The quartet of interactions between the species Smu and Sp. The growth-resource curves of Smu (A) and Sp (B) and the community, monoculture and left-over growth rates of the two species indicated (see text for meanings of the various symbols). (C) The positive (blue) and negative (red) interaction components, forming the interaction quartet, and the net (white hatched) interactions between the species.

We considered the species *Streptococcus mutans* (Smu) and *Streptococcus parasanguinis* (Sp), which are part of the human oral microbiome [30]. The (input) data required to apply our method are the growth-resource curves of the two species, $\mu_{Smu}(R)$ and $\mu_{Sp}(R)$, and estimates of their growth rates in the two-species community, $\mu_{Smu}^{c}$ and $\mu_{Sp}^{c}$, at a given resource supply rate, $R_{c}$. This would allow us to estimate the quartet at $R_{c}$. The input data could be obtained experimentally (see below). Here, for illustration, we used computational approaches. Using genome-scale metabolic reconstructions of the two species (Methods), we predicted their individual growth-resource curves. We considered resources resembling a Western diet and quantified the resource supply rate, R, as the fold-increase in the supply rate of the resources over a basal rate (Methods). The ingredients in the resource, which include sugars, amino acids, minerals and a host of other nutrients, along with their basal uptake rates are listed in S8 Table. Unless specified otherwise, R thus represents the fold-increase over the basal rate and hence is dimensionless. Further, values of R<1 would imply resource constraints, whereas R>1 would imply an enriched medium. Over the range of R we examined, the growth-resource curves were linear (Fig 2A and 2B), but with different slopes. The curve for Smu had a higher slope than Sp. Thus, at any R, the growth rate of Smu, $\mu_{Smu}$, was higher than that of Sp, $\mu_{Sp}$. For instance, at R=1, the rates were: $\mu_{Smu}\sim \text{0}.\text{5}\;h^{-\text{1}}$ and $\mu_{Sp}\sim \text{0}.\text{3}\;h^{-\text{1}}$.

We now considered a community environment with a resource supply rate $R_{c}=\text{2}$ and estimated the monoculture and community growth rates of the species (Methods). The growth rate of Smu in the coculture was $\mu_{Smu}^{c}=\text{0}.\text{5}\text{2}\text{2}\;h^{-\text{1}}$, while that of Sp was $\mu_{Sp}^{c}=\;\text{0}.\text{5}\text{0}\text{9}\;h^{-\text{1}}$. The corresponding monoculture growth rates of the species were $\mu_{Smu}^{m}=\text{0}.\text{9}\text{4}\;h^{-\text{1}}$ and $\mu_{Sp}^{m}=\text{0}.\text{6}\text{3}\;h^{-\text{1}}$. The difference between the community growth rates and the monoculture growth rates is attributed to the interactions between the species, driven both by competition for resources and beneficial exchange of cross-feeding metabolites. The list of shared nutrients is in S8 Table, whereas cross-fed metabolites are listed in S9 Table. From these estimates, we obtained the net interactions as the difference of the growth rates in the coculture and monoculture, so that $\Delta_{Smu\leftarrow Sp}^{net}=-\text{0}.\text{418}\;h^{-\text{1}}$and $\Delta_{Sp\leftarrow Smu}^{net}=\;-\text{0}.\text{121}\;h^{-\text{1}}$ (Fig 2C). The pair thus exhibited competition, with each species negatively impacting the growth of the other. With this input data, we could now apply our method and estimate the components of these net interactions.

Following the procedure above, we first identified the resource supply rate required for Smu to sustain its community growth rate in the absence of additional interactions. This yielded the resource supply rate $R_{Smu}^{c}=\;\text{1}.\text{08 }$(Fig 2A), which ensured that $\mu_{Smu}=\mu_{Smu}^{c}=\text{0}.\text{522}\;h^{-\text{1}}$ (Fig 2B). $R_{Smu}^{c}$ was less than $R_{c}=\text{2}$. This implied that the remaining resources $\Delta R_{Smu}^{c}=\text{2}-\text{1}.\text{0}\text{8}=\text{0}.\text{9}\text{2}$ were left over after sustaining Smu at its community growth rate (Fig 2A). Consequently, the left-over growth rate of Sp, $\mu_{Sp}^{l}=\mu_{Sp}\left(\Delta R_{Smu}^{c}\right)=\text{0}.\text{2}\text{8}\text{8}$ $h^{-\text{1}}$. This growth rate was less than that observed for Sp in the community, implying that Sp experienced a positive influence from Smu. The positive component of the influence of Smu on Sp was thus (Eq. (3)): $\Delta_{Sp\leftarrow Smu}^{+}=\mu_{Sp}^{c}-\mu_{Sp}^{l}=\text{0}.\text{5}\text{0}\text{9}-\text{0}.\text{2}\text{8}\text{8}=\text{0}.\text{2}\text{2}\text{1}\;h^{-\text{1}}$. The negative component followed as the difference between the net interaction and the positive component:$\Delta_{Sp\leftarrow Smu}^{-}=\Delta_{Sp\leftarrow Smu}^{net}-\Delta_{Sp\leftarrow Smu}^{+}=-\text{0}.\text{1}\text{2}\text{5}-\text{0}.\text{2}\text{2}\text{1}=-\text{0}.\text{3}\text{4}\text{6}\;h^{-\text{1}}.$

We repeated the above procedure for estimating components of the influence of Sp on Smu. The resource supply rate needed for sustaining the growth rate of Sp, $\mu_{Sp}$, at its community growth rate, $\mu_{Sp}^{c}=\;\text{0}.{\text{5}\text{0}\text{9}\;h}^{-\text{1}}$, was $R_{Sp}^{c}=\text{1}.\text{5}\text{6}\;$(Fig 2B). The resource left over after sustaining Sp at its community growth rate was ${\Delta R}_{Sp}^{c}=R_{c}-\;R_{Sp}^{c}=\text{0}.\text{4}\text{4}$. We estimated the growth rate of Smu that can be afforded by the latter left-over resource to be $\mu_{Smu}^{l}=\mu_{Smu}\left({\Delta R}_{Sp}^{c}\right)=\text{0}.\text{1}\text{9}\text{9}\;h^{-\text{1}}$ (Fig 2B). The positive component of the influence of Sp on Smu was thus $\Delta_{Smu\leftarrow Sp}^{+}=\mu_{Smu}^{c}-\mu_{Smu}^{l}=\text{0}.\text{5}\text{2}\text{2}-\text{0}.\text{1}\text{9}\text{9}=\text{0}.\text{3}\text{2}\text{3}\;h^{-\text{1}}$. The negative component followed from the net interaction: $\Delta_{Smu\leftarrow Sp}^{-}=\Delta_{Smu\leftarrow Sp}^{net}-\Delta_{Smu\leftarrow Sp}^{+}=-\text{0}.\text{4}\text{1}\text{5}-\text{0}.\text{3}\text{2}\text{3}=-\text{0}.\text{7}\text{3}\text{8}\;h^{-\text{1}}$. Our method thus yielded the quartet for the Smu-Sp pair: ${(\Delta }_{Smu\leftarrow Sp}^{+},\;\Delta_{Smu\leftarrow Sp}^{-},\Delta_{Sp\leftarrow Smu}^{+},\Delta_{Sp\leftarrow Smu}^{-})=(\text{0}.\text{3}\text{2}\text{3},\;-\text{0}.\text{7}\text{3}\text{8},\;\text{0}.\text{2}\text{2}\text{1},\;-\text{0}.\text{3}\text{4}\text{6})$ $h^{-\text{1}}$ (Fig 2C).

The example above illustrates the application of our method. We next applied it to estimate the quartets of all species pairs in a representative human oral microbiome.

### The quartets of interactions between species in a representative oral microbiome

We considered the collection of 8 species that has been employed previously to construct a synthetic human oral microbiome [12,31]: *Actinomyces viscosus* (Av), *Streptococcus mitis* (Smi), *Streptococcus salivarius* (Sl), *Streptococcus sanguinis* (Ss), *Bifidobacterium lactis* (Bf), and *Lactobacillus casei* (Lc), along with Smu and Sp. We first estimated the net pairwise interactions between the species. We employed genome-scale metabolic models of the species and estimated the monoculture and community growth rates of the species in all possible two-species communities at the resource supply rate $R_{c}=\text{2}$ (Methods; S1-S7 Tables). We employed two different methods, MICOM [32] and SteadyCom [33], for community modelling to ascertain the robustness of our findings. We present results with MICOM here. Results with SteadyCom, which were similar to those with MICOM, are in the Supplementary Materials (see S1 Text, S1 and S2 Figs, S5-S7 Tables).

**Fig 3:**
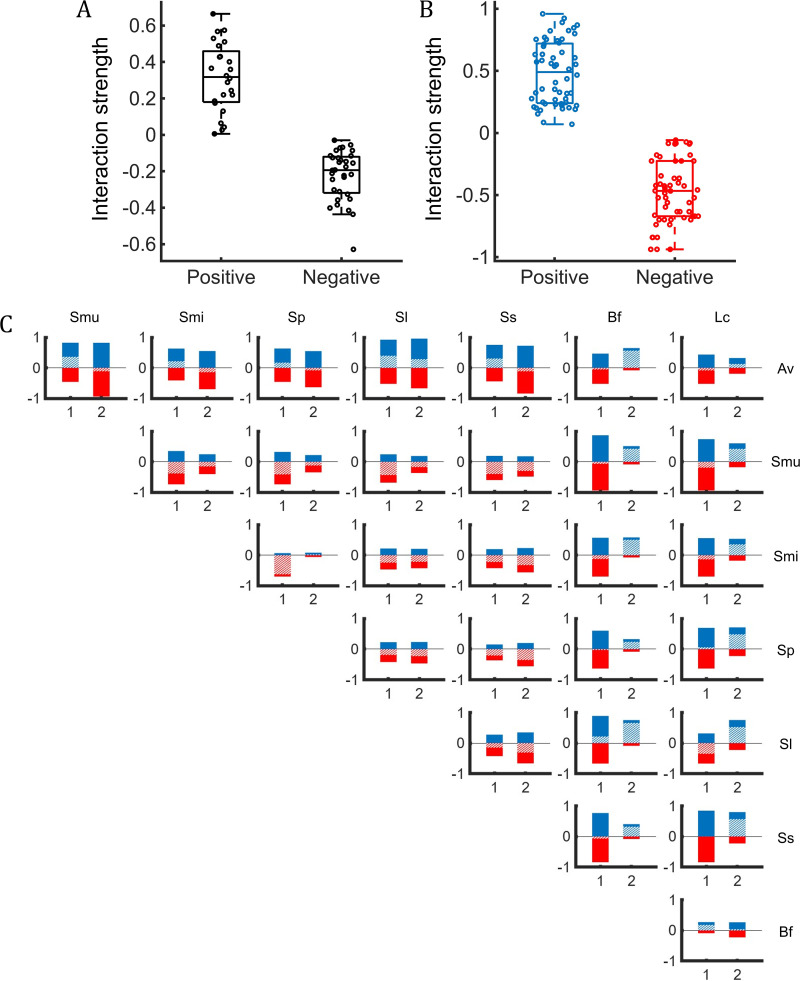
The quartets of interactions between species pairs in a representative oral microbiome. The distribution of (A) the net interactions and (B) their components between all pairs of species in a representative human oral microbiome (see text). In (A), the net positive and net negative interactions are shown separately for clarity. Boxes show median and interquartile ranges and whiskers show extremes. (C) The components (blue – positive; red – negative) and the net interactions (white hatched). In each panel, 1 is the species mentioned next to the row (row species) and 2 that above the column (column species) to which the panel belongs. The x-axis labels (1 or 2) indicate the species on which the influence of the other (2 or 1) is estimated.

Among the 28 species pairs, resulting in 56 net interactions, we found that 24 interactions were net positive and 32 interactions were net negative (Fig 3A;S3 Table). The list of shared nutrients and cross-fed metabolites are in S8 and S9 Tables, respectively. The strengths of the positive and negative interactions were comparable: the median positive interaction strength was $\text{0}.\text{3}\text{2}\;h^{-\text{1}}$ (IQR: $[\text{0}.\text{1}\text{8},\;\text{0}.\text{4}\text{4}]\;h^{-\text{1}}$), whereas the median negative interaction strength was $-\text{0}.\text{1}\text{9}\;h^{-\text{1}}$ (IQR: $[-\text{0}\mathrm{.31},\;-\text{0}\mathrm{.12}]\;h^{-\text{1}}$) (Fig 3A). Interestingly, of the 28 species pairs, 14 pairs showed exploitation or parasitic interactions, 9 competitive interactions, and 5 mutualistic interactions. This predominance of exploitation/competition and the limited occurrence of mutualism was consistent with measurements on culturable bacterial species [34].

We next estimated the components of these net interactions using our method above. We exploited the linear dependence of the growth rates on the resource supply rate (see Fig 2A) to estimate the components without explicitly computing the entire growth-resource curves (Methods). Of the 112 components (constituting the 56 net interactions), we found all the components to be non-zero, indicating that the net interactions were the resultants of underlying positive and negative components (Fig 3B and 3C;S4 Table). Overall, the positive and negative components estimated were similarly powerful: The median positive component was $\text{0}.\text{4}\text{9}\;h^{-\text{1}}\;$(IQR: $[\text{0}.\text{2}\text{4},\;\text{0}.\text{7}\text{1}]h^{-\text{1}}$) and the median negative component was $-\text{0}.\text{4}\text{7}\;h^{-\text{1}}$ (IQR: $[-\text{0}\mathrm{.67},\;-\text{0}\mathrm{.23}]\;h^{-\text{1}}$) (Fig 3B).

Interestingly, we observed that species pairs existed with similar net interactions but vastly different underlying components. For instance, the Av-Bf pair had similar net interactions to the Smu-Bf pair: ${(\Delta }_{Av\leftarrow Bf\;}^{net},\;\Delta_{Bf\leftarrow Av}^{net})=(-\text{0}.\text{0}\text{5}\text{5},\;\text{0}.\text{5}\text{6}\text{8})\;h^{-\text{1}}$ and ${(\Delta }_{Smu\leftarrow Bf\;}^{net},\;\Delta_{Bf\leftarrow Smu}^{net})=(-\text{0}.\text{0}\text{6}\text{8},\;\text{0}.\text{4}\text{3})\;h^{-\text{1}}$. The components, however, were quite different in magnitude between the pairs. The quartets were: ${(\Delta }_{Av\leftarrow Bf\;}^{+},\;\Delta_{Av\leftarrow Bf\;}^{-},\;\Delta_{Bf\leftarrow Av}^{+},\;\Delta_{Bf\leftarrow Av}^{-})=(\text{0}.\text{466},-\text{0}.\text{522},\;\text{0}.\text{649},-\text{0}.\text{084}){\;h}^{-\text{1}}\;$and ${(\Delta }_{Smu\leftarrow Bf\;}^{+},\Delta_{Smu\leftarrow Bf\;}^{-},\;\Delta_{Bf\leftarrow Smu}^{+},\;\Delta_{Bf\leftarrow Smu}^{-})=(\text{0}.\text{8}\text{6}\text{8},\;-\text{0}.\text{9}\text{3}\text{7},\;\text{0}.\text{5}\text{1}\text{3},\;-\text{0}.\text{0}\text{8}\text{4})\;h^{-\text{1}}$. The existence of such pairs highlights the importance of the quartet. The two pairs are likely to behave differently when subjected to the same environmental changes, a distinction that extant theories, reliant on net interactions alone, may not capture.

Further, we recognized that small net interactions could arise from the cancellation of strong underlying components. For instance, Av had a weak net negative influence on Ss, with $\Delta_{Ss\leftarrow Av}^{net}=-\text{0}.\text{1}\text{1}\text{6}\;h^{-\text{1}}$. However, this small net negative interaction was made up of large positive and negative components: $\Delta_{Ss\leftarrow Av}^{+}=\text{0}.\text{7}\text{2}\text{5}\;h^{-\text{1}}$ and $\Delta_{Ss\leftarrow Av}^{-}=-\text{0}.\text{8}\text{4}\text{1}\;h^{-\text{1}}$. Similarly, in the same species pair, Ss had a net positive effect on Av, with $\Delta_{Av\leftarrow Ss}^{net}=\text{0}.\text{3}\text{1}{\;h}^{-\text{1}}$, but this net effect arose from strong components: $\Delta_{Av\leftarrow Ss}^{+}=\text{0}.\text{7}\text{5}\text{3}\;h^{-\text{1}}$, and $\Delta_{Av\leftarrow Ss}^{-}=\;-\text{0}.\text{4}\text{4}\text{3}\;h^{-\text{1}}$. The species thus exhibited strong influences on each other; weak net interactions need not imply weak components. Extant theories predict that weak net interactions are stabilizing [7,35]. Whether the predictions hold when the underlying components are strong remains to be assessed.

These observations make a case for using the quartet to characterize species interactions. Next, we examined the implications of the quartet for understanding the behaviour of communities.

### Implications of the quartet for understanding community behaviour

The above predictions were made at a single community resource supply rate ($R_{c}=\text{2}$). We next examined how the quartet would change as the resource supply rate is varied. We considered the pair of Av and Bf, discussed above. Recall that at $R_{c}=\text{2}$, the pair exhibited exploitation. We varied $R_{c}$ over a wide range, from 2 to 10. At each value of $R_{c}$ in this range, we applied our procedure above and estimated the net interactions as well as the quartet.

**Fig 4:**
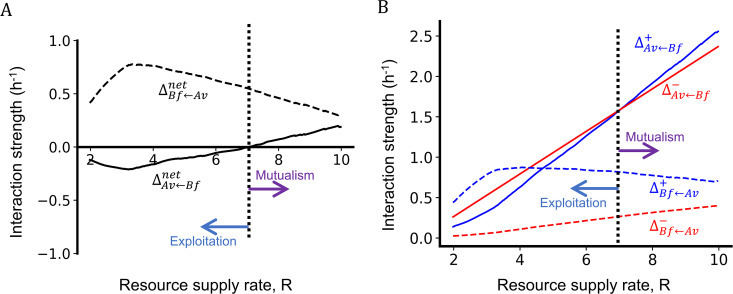
Variation of the quartet with resource availability for the Av-Bf pair. The variation of (A) the net interactions and (B) the quartet components of the Av-Bf pair with the resource supply rate. Solid lines indicate the influence of Bf on Av and dashed lines of Av on Bf. Positive components are in blue and negative in red. The vertical dotted line marks the transition from exploitation to mutualism with increasing resource supply. The magnitudes of the negative components are plotted for easy comparison with their positive counterparts.

The net interactions displayed complex non-monotonic trends, with the pair transitioning from exploitation to mutualism with increase in $R_{c}$ (Fig 4A). Specifically, Bf exerted a net negative influence on Av, which decreased (became more negative) with $R_{c}$ until 3.5, after which it increased. Eventually, beyond $R_{c}\sim \text{7}$, the interaction changed sign with Bf exerting a positive influence on Av. Av, on the other hand, exhibited a net positive influence on Bf throughout, although the strength of this interaction increased until $R_{c}\sim \text{3}.\text{5}$ and then decreased. Thus, at $R_{c}\sim \text{7}$, the community switched from exploitation to mutualism. How such complex trends emerged with increasing $R_{c}$ is puzzling. We understood this using the quartet.

We found, interestingly, that all the components of the quartet varied much less sharply with $R_{c}$ than the net interactions (Fig 4B). The variation of the components relative to each other, however, was more complex and strongly affected by changes in $R_{c}$, which in turn influenced the net interactions, introducing transitions in community behaviour. As $R_{c}$ increased from 2 to ~3.5, the negative component of the influence of Bf on Av, $\Delta_{Av\leftarrow Bf}^{-}$, increased more than its positive counterpart, $\Delta_{Av\leftarrow Bf}^{+}$. (Note that here and in such comparisons throughout, we refer to the magnitudes of the components, ignoring their signs.) The net influence of Bf on Av thus became increasingly negative (Fig 4A). In contrast, over the same range of $R_{c}$, the positive component of the influence of Av on Bf, $\Delta_{Bf\leftarrow Av}^{+}$, was larger and increased more steeply with $R_{c}$ than its negative counterpart, $\Delta_{Bf\leftarrow Av}^{-}$ (Fig 4B). The net influence of Av on Bf thus became increasingly positive (Fig 4A). Consequently, the pair became more strongly exploitative as $R_{c}$ increased from 2 to ~3.5.

This trend reversed beyond $R_{c}\sim \text{3}.\text{5}$. Between $R_{c}\sim \text{3}.\text{5}$ and $R_{c}\sim \text{7}$, $\Delta_{Av\leftarrow Bf}^{-}$ remained higher than $\Delta_{Av\leftarrow Bf}^{+}$, and the latter increased more steeply with $R_{c}$ than $\Delta_{Av\leftarrow Bf}^{-}$ (Fig 4B). The net influence of Bf on Av thus became increasingly less negative after having peaked at $R_{c}\sim \text{3}.\text{5}$ (Fig 4A). However, $\Delta_{Bf\leftarrow Av}^{-}$ continued to increase with $R_{c}$, while $\Delta_{Bf\leftarrow Av}^{+}$ started gently decreasing after $R_{c}\sim \text{3}.\text{5}$ (Fig 4B), so that the net influence of Av on Bf began to decrease although the interaction remained positive (Fig 4A). Thus, the overall interaction became less exploitative and approached amensalism.

Interestingly, beyond $R_{c}\sim \text{7}$, $\Delta_{Av\leftarrow Bf}^{+}$ exceeded $\Delta_{Av\leftarrow Bf}^{-}$, so that the net influence of Bf on Av switched from being negative to positive (Fig 4A). Increasing $R_{c}$ beyond ~7 increased the extent of the positive interaction. Throughout, the influence of Av on Bf stayed positive ($\Delta_{Bf\leftarrow Av}^{+}$ started decreasing gently while $\Delta_{Bf\leftarrow Av}^{-}$ increased monotonically) and decreased its magnitude with $R_{c}$. Thus, the overall interaction now switched from exploitation to mutualism upon increasing $R_{c}$ beyond ~7.

In summary, the species pair transitioned from weak to strong exploitation, back to weak exploitation bordering on amensalism, and then to mutualism as $R_{c}$ increased from 2 to 10. These predictions illustrate how depending on the extent of increase in resources, an increase in either positive or negative interactions and hence the extent of cooperation or mutualism can result between the same species, dictated by the differential variation of the quartet components. Remarkably, the components themselves displayed smooth, gentle variations with the resources. Yet, their differential variation led to these dramatic changes in the overall interactions. This general behaviour was also evident with other species pairs we examined (S3 Fig).

We examined next whether the quartet would similarly help better understand the behaviour of communities observed experimentally.

### Application to experimental data on auxotrophs

We considered the study by Hoek *et al.* [22], which employed two engineered auxotrophs of the yeast *Saccharomyces cerevisiae*, one incapable of producing tryptophan but overexpressing leucine and the other incapable of producing leucine but overexpressing tryptophan. Auxotrophs occur widely in natural microbiomes, enriching their environments through their metabolic secretions, and may have implications for drug resistance [36]. To our study, auxotrophic pairs like the one studied by Hoek *et al.* [22] offer an excellent system because they experience both positive and negative interactions simultaneously, rendering them particularly amenable to analysis using the quartet.

**Fig 5:**
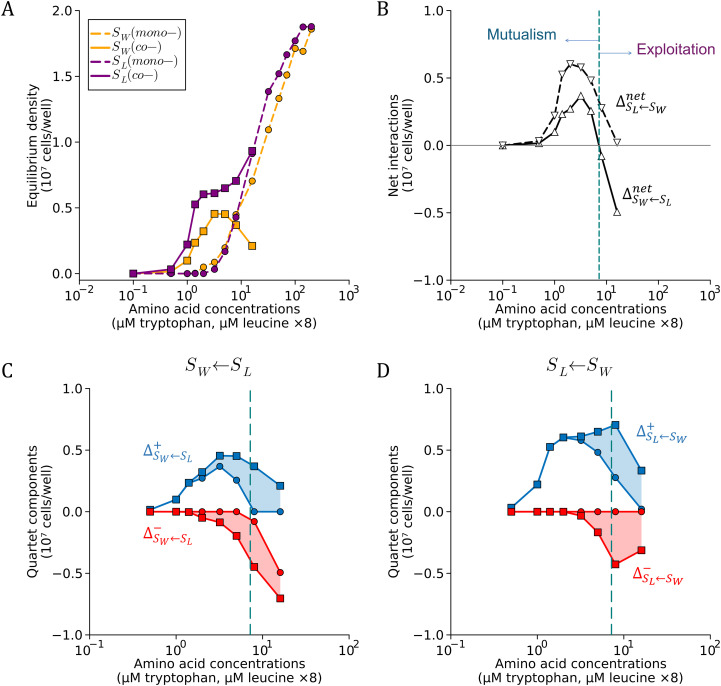
Application to a synthetic community of auxotrophs. (A) Data of the steady state abundances of the $S_{W}$ and $S_{L}$ auxotrophs in monoculture and co-coculture as functions of the supplied nutrients, reported in Hoek et al. [22]. (B) The corresponding net interactions, highlighting the regimes of mutualism and exploitations. (C) and (D) The quartet of interaction components estimated by our method, assuming the supplied amino acids being limiting (circles) or not (squares) (see Methods). Note that here the equilibrium density in (A) is used as a measure of growth rate, as it reflects the cells accrued per passage. Thus, the interaction terms in (B)-(D), which are based on differences in growth rates, have units of cell densities.

Hoek *et al.* [22] studied the auxotrophs in the following way. The species were cultured alone or together in a serial passage set-up. A 10-fold dilution was used to transfer the community population at each passage. The two amino acids, tryptophan and leucine, were replenished along with other nutrients at each passage. Passages were performed daily and each experiment ran for 7 days. A 1:8 ratio was used for tryptophan and leucine, reflecting their relative usage by the strains, and the amounts added in each passage were fixed for an experiment. In most experiments, by day 7, the abundances of the species in the cultures just before passaging reached constant values (steady state). In other words, the abundances were such that the 10-fold diluted population recovered fully (grew 10-fold) in 24 hours. These steady state abundances were reported by the authors (Fig 5A) and used in their analysis. (The steady states were not achieved with co-cultures when the amino acid supply rates exceeded 16 µM per passage; the authors then used modelling to estimate the steady state abundances.)

Based on whether the abundances in coculture were greater or smaller than the corresponding monoculture abundances, the authors inferred the net interactions between the species (Fig 5B). A higher abundance in coculture implied a net positive interaction experienced. Interestingly, the study observed multiple transitions in community behaviour, starting from unviable populations at low supply rates to mutualism, parasitism, and competition as the supply rates increased, and culminating in exclusion of the intrinsically less fit species at the highest supply rates. Here, we asked whether the quartet could help better understand these transitions.

We denoted the strains studied by Hoek *et al.* [22] as $S_{W}$ and $S_{L}$, respectively, with the subscript representing the amino acid for which the strains are auxotrophic. The monoculture data yielded the growth-resource curves necessary for our analysis (Fig 5A). We assumed that the steady state abundances were a good measure of the growth rates, as they resulted from the growth of the species in one passage. We restricted our analysis to resource supply rates up to 16 µM per passage, where experimentally observed (and not inferred) abundances were reported (Fig 5). Based on the net interactions, in this range of resource supply rates, the community witnessed a transition from mutualism to parasitism (Fig 5B). (For simplicity, we ignored the distinctions between the different types of mutualism, namely, obligate and facultative.) We applied our method to the resulting data to estimate the components.

We adapted our method to the experimental setting in two ways. First, we recognized that at low supply rates of the amino acids, the amino acids were likely to be limiting, whereas at high supply rates, other constituents of the nutrient medium were likely to be limiting (as also recognized by Hoek *et al.* [22]). Because the supply rates over which the amino acids were limiting were not known, we considered both scenarios–amino acids being limiting versus other resources being limiting–to obtain a range of values of the components. Second, the supplied amino acids were not shared between the auxotrophs, whereas other limiting nutrients were shared. Thus, the left-over resource had to be estimated differently when the amino acids were limiting (Methods).

We found that at low amino acid supply rates, the interactions were weak and so were the components (Fig 5B–5D). Gradually, the positive components, $\Delta_{S_{W}\leftarrow S_{L}}^{+}$ and $\Delta_{S_{L}\leftarrow S_{W}}^{+}$, rose because each auxotroph survived due not only to the nutrient supply but also amino acids produced by the other auxotroph. The exchange of the latter amino acids introduced the positive components. The positive components increased with the supply rate. At these low supply rates (<2 µM per passage), no negative components were observed. This is because the amino acids supplied, which were the limiting resources, were not shared. Furthermore, the components estimated assuming the two scenarios, amino acids versus some unknown nutrient being limiting, overlapped, implying, reassuringly, that the amino acids were indeed limiting when their supply rates were low.

Beyond supply rates of 2 µM per passage, negative components, $\Delta_{S_{W}\leftarrow S_{L}}^{-}$ and $\Delta_{S_{L}\leftarrow S_{W}}^{-}$, began to be manifested. Their strength increased with resource supply rates. Simultaneously, the positive components too increased until supply rates of ~5 µM per passage. This is due to the increased cross-feeding of the auxotrophic amino acids, notwithstanding the competition for other nutrients. The increase in both the positive and the negative components with the resource supply rate was consistent with our predictions above (Fig 4). Beyond ~5 µM per passage, however, the positive components began to decline. We expect this because as the supply rates of the amino acids increased, beyond a point the room for cross-feeding would decrease, a further sign that the amino acids began to be less limiting. Interestingly, the positive and negative components were not symmetric. Specifically, $\Delta_{S_{W}\leftarrow S_{L}}^{+}<\Delta_{S_{L}\leftarrow S_{W}}^{+}$ and $\Delta_{S_{W}\leftarrow S_{L}}^{-}>\Delta_{S_{L}\leftarrow S_{W}}^{-}$. (We reiterate that we compare magnitudes, ignoring signs.) In other words, the $S_{L}$ auxotroph gained disproportionately from the interactions. It gained more from cross-feeding amino acids and was hurt less from the competition for other shared nutrients than the $S_{W}$ auxotroph. Consequently, the abundance of $S_{L}$ was higher in coculture than $S_{W}$ (Fig 5A). Indeed, the abundance of $S_{W}$ began to decrease upon increasing supply rates above ~3 µM per passage. The negative component began to increase in magnitude more than the positive one. Thus, although the net interactions, $\Delta_{S_{W}\leftarrow S_{L}}^{net}$ and $\Delta_{S_{L}\leftarrow S_{W}}^{net}$, remained positive, implying mutualism, signatures of the ensuing parasitism were evident in the components. At supply rates above ~8 µM per passage, the abundance of $S_{W}$ dropped below its monoculture abundance (Fig 5A). In other words, $\Delta_{S_{W}\leftarrow S_{L}}^{net}<\text{0}$. Yet, the gain of the $S_{L}$ strain remained net positive, $\Delta_{S_{L}\leftarrow S_{W}}^{net}>\text{0}$, marking parasitism. Throughout, with mutualism or parasitism, as the abundance of $S_{W}$ decreased, the abundance of $S_{L}$ increased. The rate of increase, however, was lower than in monoculture, signifying the effect of the negative influence of $S_{W}$ on $S_{L}$. Indeed, at supply rates of ~16 µM per passage, the abundance of $S_{L}$ became similar to that in monoculture, indicating amensalism (Fig 5A and 5B). The quartet estimates show that this transition too is a manifestation of the differential changes in the components despite their smooth dependence on the resource supply rate (Fig 5C and 5D). The quartet thus helped better understand the community behaviour, including underlying transitions, and offered insights beyond those from net interactions.

## Discussion

Recognizing that species can engage in both positive and negative interactions simultaneously [11,17,21,22,24], efforts are underway to devise methods to estimate these interaction components. Computational approaches including those based on genome-scale metabolic models offer qualitative insights into the components [17,23,25,26]. KIDI, a method that marks the most recent advance, helps estimate the components quantitatively [27]. It is restricted, however, to specialized settings where cross-feeding metabolites and shared limiting nutrients are explicitly known along with their kinetic relationships with species growth rates. A more widely applicable method, to our knowledge, is not available. The conventional characterization of species interactions, based on the net interactions, has therefore prevailed, often limiting our understanding of the behaviour of multispecies communities and ability to design and engineer them. Here, we present a new method to estimate the positive and negative components of the net interactions, yielding the quartet of components governing species interactions. The method requires as inputs the growth-resource curves of the species involved and their community growth rates, which are all more readily obtained than the data on which extant approaches rely. Specifically, it does not require knowledge of specific cross-feeding metabolites or shared nutrients. It then estimates the components using a conceptual resource partitioning strategy which is also easy to implement. We expect our method, therefore, to have wide applicability.

We applied our method to several *in silico* species pairs and an experimental auxotrophic pair. Several insights emerged that have implications for our understanding of multispecies communities. We found that species pairs could exist with similar net interactions but vastly different underlying components. This finding makes evident the limitation of characterizing species interactions using net interactions. One expects such species pairs to behave differently when subjected to given environmental stresses. Current community ecology theories, reliant on net interactions alone, would predict in contrast that the pairs would behave similarly. We also found that weak net interactions could arise from the cancellation of strong underlying components. Current theories predict that weak net interactions are stabilizing [7,35]. Whether weak net interactions remain stabilizing despite strong underlying components remains to be ascertained. We anticipate the development of new, more refined community ecology theories based on the quartet that would capture these nuances.

The insights from the application of our method extended to transitions in community behaviour upon altering resource availability. Increasing resource availability is argued to minimize the need for cooperation, thereby increasing competition between species pairs [9,24,34]. Yet, several instances exist where cooperation has been observed to increase with increasing resource availability [21,22]. Attempts are being made to reconcile these conflicting observations. For instance, the extent of cooperation and competition have been attributed not only to the availability of resources but also to the extent of overlap in the utilization of resources, with limited overlap enhancing cooperation with increasing resource supply [21,37]. Costless metabolites, which incur minimal production costs but can be cross-fed [38], may also increase cooperation with increasing resource availability. Our calculations using the quartet offer a more general reconciliation. As resource availability increases, although the need for cooperation may decrease, the production of cross-feeding metabolites, which are products of the metabolism of the resources, may increase, enhancing cooperation. Thus, whether cooperation or competition increases will depend on how the underlying positive and negative components change relative to each other with increasing resources. Indeed, we found that all the components, positive and negative, changed gradually upon increasing resource availability. It was their relative variation that manifested as complex changes in the net interactions and hence community behaviour, both *in silico* and *in vitro* [22].

The application of our method to the experimental auxotrophic pair [22], which also showed that the quartet could be estimated using data that can be obtained experimentally, brought out nuances in the behaviour of the community that went beyond those anticipated based on the net interactions alone. In the original study, Hoek *et al.* [22] developed a mathematical model incorporating empirically the cooperative effect of the auxotrophs producing the complementary amino acids and the competitive effects arising from the sharing of other (unspecified) resources, which gave rise to the competition when amino acid supplies were abundant. The model qualitatively recapitulated the transitions in community behaviour observed. Our analysis offered further insights. It showed that the quartet carried signatures of impending transitions well before the transitions occurred. It also showed when the auxotrophs shifted from being limited by the supplied amino acids to competing for the other supplied nutrients. Further, it brought out the asymmetry in the benefit accrued by each auxotroph from the amino acid secreted by the other. Importantly, these insights emerged without the need for dynamical modelling, such as the use of Lotka-Volterra-type equations as has been done previously [22,27], and the estimation of associated parameters from the data. Thus, these insights, which were present cryptically in the data, were revealed by the application of our method to estimate the quartet components. Given the diverse environments in which auxotrophs exist [36], such insights may prove valuable beyond the specific experimental system we considered, aiding the description of the associated ecosystems.

The *in silico* species we considered belong to the oral microbiome, the behaviour of which is yet to be fully understood. Studies have observed that the natural oral community exhibits complex spatio-temporal organization [39]. Attempts have been made to describe representative synthetic oral communities using net interactions [12]. Yet, that the underlying positive and negative components may be important to understand the complexities involved has been recognized [39]. Earlier estimates of the components have remained qualitative, resorting to measures such as the fractions of metabolites shared between species [26,39]. Here, we offer a more accurate, quantitative estimation of the components. Incorporating them in models of spatio-temporal pattern formation [40], a promising avenue for future research, may help better describe the complex dynamics of the oral microbial community.

The relative frequency of competitive and cooperative interactions between species has been actively investigated in recent years [7,8,10,24,34,41–48]. On one side, evidence exists that cooperative interactions improve the efficiency of the microbial community, by distributing the metabolic burden between species or enabling new collective functionalities [41–43]. Cooperation has been seen, for instance, between species in the gut microbiome and between species grown under extreme, resource starved conditions [24,44]. Challenging this view, ecological theory argues that cooperative interactions may render communities unstable [7,15]. Competitive interactions, in contrast, are predicted to be stabilizing. In agreement, experiments on culturable bacteria from several niches have found a predominance of competitive interactions between species [8,10,34,45–48]. A comprehensive understanding of the factors determining the predominance of competition versus cooperation is still lacking. Here, consistently with experiments, we estimated that competitive interactions were predominant in the *in silico* oral microbial community we studied. Future studies may assess whether the strengths of the components we estimated were also reflected in the wider set of culturable species.

We recognize limitations of our study. First, it is restricted to scenarios dominated by metabolic interactions, wherein, in particular, negative interactions arise from shared resources. While such interactions are the more common ones [17–22], scenarios where other types of interactions, driven for instance by toxins or signalling molecules [49], dominate may find behaviours distinct from those predicted by our study. Second, our method yields upper bounds on the magnitudes of the components. By allotting one of the species all the resources necessary for its sustenance in the community and letting the other species be sustained by left-over resources and positive interactions, the latter interactions may be overestimated. Third, we recognize that genome-scale metabolic models may not always capture the interactions between species accurately [17,31]. For instance, genome-scale models correctly predicted observed shifts between negative and positive interactions between species upon changing media conditions in only 65% of the cases [17]. Therefore, although we ascertained the robustness of our findings by using two different community modelling approaches, our findings based on the models remain conceptual. Our method, though, is not limited by the models. It can be implemented using data generated experimentally, bypassing genome-scale and community models entirely, as we demonstrated with our analysis of the two-auxotroph community. Fourth, our method does not consider potential catabolite repression schemes [50], which may affect our predictions if, for instance, cross-feeding metabolites affect substrate utilization priorities due to such repression schemes. Finally, our study is restricted to pair-wise interactions, whereas high-order interactions may exist in multispecies communities [11,12,31,51,52]. Advancing the quartet to high-order interactions, often necessary to predict community behaviours [11,12,51,52], would be an important future goal.

In conclusion, we present a broadly applicable method to disentangle the positive and negative influences of species on each other in a community. The resulting quartet of interaction components is a more fundamental and more nuanced characterization of species interactions than the prevalent, conventional paradigm using the net interactions alone. The ability to estimate the quartet would prompt the development of more refined theories of microbial community ecology that would enable better understanding of the behaviour of multispecies communities and elucidate more robust principles for their design and engineering.

## Methods

### Estimation of the quartet

Here, we summarize our procedure for estimating the quartet of interactions between two species, denoted 1 and 2. Derivation of the procedure is in Results. We consider a community environment with resource supply rate $R_{c}$. The procedure that follows allows estimation of the quartet at $R_{c}$. The input data required are the growth rates of the two species in the community environment, denoted $\mu_{\text{1}}^{c}$ and $\mu_{\text{2}}^{c}$, and the growth-resource curves of the two species in monoculture, denoted $\mu_{\text{1}}(R)$ and $\mu_{\text{2}}(R)$, respectively. Recall that the growth-resource curves represent the variations of the growth rates of the species, $\mu_{\text{1}}$ and $\mu_{\text{2}}$, with the resource supply rate, R. With this input data, we first find the monoculture growth rates of the two species at the resource supply rate mimicking the community environment. In other words, we find $\mu_{\text{1}}^{m}=\mu_{\text{1}}(R_{c})$ and $\mu_{\text{2}}^{m}=\mu_{\text{2}}(R_{c})$. The difference between the community and monoculture growth rates yields the net interactions: $\Delta_{\text{1}\leftarrow \text{2}}^{net}=\mu_{\text{1}}^{c}-\mu_{\text{1}}^{m}$ and $\Delta_{\text{2}\leftarrow \text{1}}^{net}=\mu_{\text{2}}^{c}-\mu_{\text{2}}^{m}$. Next, we find the positive components of the net interactions as follows. From the growth-resource curves, we find the resource supply rates, denoted $R_{\text{1}}^{c}$ and $R_{\text{2}}^{c}$, respectively, at which the monoculture growth rates of the species would equal their corresponding community growth rates. In other words, we find $R_{\text{1}}^{c}$ as that value of R that satisfies $\mu_{\text{1}}(R)=\mu_{\text{1}}^{c}$. Similarly, we find $R_{\text{2}}^{c}$ as that value of *R* that satisfies $\mu_{\text{2}}(R)=\mu_{\text{2}}^{c}$. From these supply rates, we estimate the left-over resources, $\Delta R_{\text{1}}^{c}=R_{c}-R_{\text{1}}^{c}$ and $\Delta R_{\text{2}}^{c}=R_{c}-R_{\text{2}}^{c}$, as the resources in the community in excess of those required for the individual species to grow at their respective community growth rates in the absence of any interactions. These left-over resources are then used to estimate the left-over growth rates, $\mu_{\text{1}}^{l}=\mu_{\text{1}}(\Delta R_{\text{2}}^{c})$ and $\mu_{\text{2}}^{l}=\mu_{\text{2}}(\Delta R_{\text{1}}^{c})$, as the growth rates of each species afforded by the resources left-over by the other species. The difference between the community growth rates and the corresponding left-over growth rates yields estimates of the positive components: $\Delta_{\text{1}\leftarrow \text{2}}^{+}=\mu_{\text{1}}^{c}-\mu_{\text{1}}^{l}$ and $\Delta_{\text{2}\leftarrow \text{1}}^{+}=\mu_{\text{2}}^{c}-\mu_{\text{2}}^{l}$. Finally, we estimate the negative components as the difference between the net interactions and the corresponding positive components: $\Delta_{\text{1}\leftarrow \text{2}}^{-}=\Delta_{\text{1}\leftarrow \text{2}}^{net}-\Delta_{\text{1}\leftarrow \text{2}}^{+}$ and $\Delta_{\text{2}\leftarrow \text{1}}^{-}=\Delta_{\text{2}\leftarrow \text{1}}^{net}-\Delta_{\text{2}\leftarrow \text{1}}^{+}$. This yields the quartet of components at $R_{c}$: $\Delta_{\text{1}\leftarrow \text{2}}^{+}$, $\Delta_{\text{1}\leftarrow \text{2}}^{-}$, $\Delta_{\text{2}\leftarrow \text{1}}^{+}$, and $\Delta_{\text{2}\leftarrow \text{1}}^{-}$. If the resource supply rate, $R_{c}$, is changed, the above procedure must be followed at the new supply rate to estimate the quartet applicable at that rate.

### Species metabolic models

We applied our method to species pairs from a representative oral microbial community [31], comprising the 8 species listed above (see Results). The community has been experimentally assembled and studied as a synthetic mimic of the human oral microbiome [12,31]. We retrieved their metabolic models from the Virtual Metabolic Human database [53,54] (VMH database), which were curated semi-automatically. The draft reconstructions of the metabolic models were curated based on available knowledge and genomic evidence. These reconstructions are state-of-the-art, publicly available metabolic models for the microbes we considered.

### Nutrient medium

The nutrient medium we selected followed the Western diet [53]. The components of the medium are shown in S8 Table. We identified the set of common exchange reactions between the species involved and let it constitute the base medium for the species. To make the nutrient medium richer or poorer relative to the base medium, we multiplied the maximum uptake rates in the base medium by a fixed factor. For instance, when the factor was 2, the medium had maximum uptake rates twice those in the base medium.

### Community modelling

We used MICOM [32] for modelling cocultures, which is implemented in Python. For robustness, we also used SteadyCom [33], which we implemented using the createMultiSpeciesModel and SteadyCom functions of the COBRA Toolbox [55] to create and analyze metabolic models of cocultures, respectively. Microbiomes usually exist under balanced growth conditions [33], which is characterized by equal specific growth rates of all species, also called the community growth rate, $\mu_{c}$. SteadyCom guarantees the balanced growth of species in the community [33]. The method employs the nutrient supply rates and computes the community growth rate, $\mu_{c}$, and the species compositions (i.e., their relative abundances). From the latter, the growth rates of the individual species are estimated in the community as follows. If $x_{i}$ were the relative abundance of species i, then its ‘fractional’ growth rate, or its relative contribution to the community growth rate, would be $\mu_{c}x_{i}$, which we used as a proxy for the species growth rate, $\mu_{i}^{c}$. MICOM also considers balanced growth but estimates the growth rates, $\mu_{i}^{c}$, using an alternative heuristic approach that leads to realistic growth rate estimates in multispecies communities [32]. In particular, it allows the specific growth rates of the species to deviate from the balanced growth condition. The optimization problems solved by MICOM and SteadyCom are stated in S2 Text.

### Quartet simplified for linear growth-resource curves

The relationship between the growth rate, μ, and the limiting resource supply rate, R, is often linear. The saturation of the growth rate in Monod kinetics [29] implies that the resource is no longer limiting. Nonetheless, when the relationship is linear, it allows the application of our method without the need to explicitly construct the growth-resource curves. We present this simplified method here. Linear dependence would imply that the growth rates of the two species may be written as $\mu_{\text{1}}=a_{\text{1}}R$ and $\mu_{\text{2}}=a_{\text{2}}R$, where $a_{\text{1}}$ and $a_{\text{2}}$ represent the respective proportionality constants. We first estimate the monoculture and community growth rates under the given resource supply setting, say $R_{c}$. By definition, $\mu_{\text{1}}^{m}=a_{\text{1}}R_{c}$ and $\mu_{\text{2}}^{m}=a_{\text{2}}R_{c}$. Let the growth rates in the community be $\mu_{\text{1}}^{c}$ and $\mu_{\text{2}}^{c}$, respectively. Again, by definition, $\mu_{\text{1}}^{c}=a_{\text{1}}R_{\text{1}}^{c}$ and $\mu_{\text{2}}^{c}=a_{\text{2}}R_{\text{2}}^{c}$. Recall that $R_{\text{1}}^{c}$ and $R_{\text{2}}^{c}$ are the resource supply rates required to sustain the growth rates of species 1 and 2, respectively, in the community in the absence of any interactions. The left over resource supply rates are therefore $\Delta R_{\text{1}}^{c}=R_{c}-R_{\text{1}}^{c}$ and $\Delta R_{\text{2}}^{c}=R_{c}-R_{\text{2}}^{c}$. Again, using linearity, we obtain the left-over growth rates, $\mu_{\text{1}}^{l}=a_{\text{1}}\Delta R_{\text{2}}^{c}$ and $\mu_{\text{2}}^{l}=a_{\text{2}}\Delta R_{\text{1}}^{c}$. We combine these relations to obtain expressions for the left-over growth rates. Thus, $\mu_{\text{1}}^{l}=a_{\text{1}}\Delta R_{\text{2}}^{c}=\left(\frac{\mu_{\text{1}}^{m}}{R_{c}}\right)\times \left(R_{c}-R_{\text{2}}^{c}\right)=\left(\frac{\mu_{\text{1}}^{m}}{R_{c}}\right)\times \left(R_{c}-\left(\frac{\mu_{\text{2}}^{c}}{a_{\text{2}}}\right)\right)=\left(\frac{\mu_{\text{1}}^{m}}{R_{c}}\right)\times \left(R_{c}-\left(\frac{\mu_{\text{2}}^{c}}{\mu_{\text{2}}^{m}}\right)R_{c}\right)=\mu_{\text{1}}^{m}\left(\text{1}-\frac{\mu_{\text{2}}^{c}}{\mu_{\text{2}}^{m}}\right).\;$In the same way, $\mu_{\text{2}}^{l}=\mu_{\text{2}}^{m}\left(\text{1}-\frac{\mu_{\text{1}}^{c}}{\mu_{\text{1}}^{m}}\right)$. With the left-over growth rates identified, the interactions follow as described in the Results (see Figs 1 and 2). Note that if the linearity of the dependence of the growth rate on resource supply rates is violated, then the full growth-resource curves would have to be estimated. The procedure outlined in the Results (Fig 1) for the estimation of the components then follows.

The growth-resource curves above, namely, $\mu_{\text{1}}=a_{\text{1}}R$ and $\mu_{\text{2}}=a_{\text{2}}R$, can be readily modified to account for ATP maintenance requirements. Consider the minimum resource levels, $R_{\text{1}}^{min}$ and $R_{\text{2}}^{min}$, respectively, required for maintenance. The growth-resource curves could then be written as: $\mu_{\text{1}}=a_{\text{1}}(R-R_{\text{1}}^{min})$ and $\mu_{\text{2}}=a_{\text{2}}(R-R_{\text{2}}^{min})$. This ensures that no growth is possible below the minimum resource levels. In the community environment, in the pure competition setting, it follows that neither species would grow at resource levels below $R_{c}^{min}=R_{\text{1}}^{min}+R_{\text{2}}^{min}$. Thus, when $R_{c}>R_{c}^{min}$, we follow the procedure above with the rescaled resource supply rates, $R_{\text{1}}^{\prime }=R_{\text{1}}-R_{\text{1}}^{min}$ and $R_{\text{2}}^{\prime }=R_{\text{2}}-R_{\text{2}}^{min}$, and estimate the quartet.

### Application to auxotrophs

Our method can be readily applied to auxotrophs, which lack the capability to synthesize specific metabolites essential for their survival. When the metabolites are not supplied in the nutrient medium, the auxotrophs can only grow in the presence of other species that can synthesize those metabolites. We consider a two-species community with species 1 an auxotroph for a metabolite produced by species 2 but not part of the nutrient medium. Thus, $\mu_{\text{1}}^{m}=\text{0}$, at all resource supply rates. The net interaction is therefore, $\Delta_{\text{1}\leftarrow \text{2}}^{net}=\mu_{\text{1}}^{c}-\mu_{\text{1}}^{m}=\mu_{\text{1}}^{c}$. Because the nutrient medium lacks the essential metabolite, left-over resources too cannot contribute to the growth of species 1, implying that $\mu_{\text{1}}^{l}=\text{0}$. Thus, $\Delta_{\text{1}\leftarrow \text{2}}^{+}=\mu_{\text{1}}^{c}-\mu_{\text{1}}^{l}=\mu_{\text{1}}^{c}$, reiterating the notion that species 1 may only grow due to the positive influence of species 2. Finally, $\Delta_{\text{1}\leftarrow \text{2}}^{-}=\Delta_{\text{1}\leftarrow \text{2}}^{net}-\Delta_{\text{1}\leftarrow \text{2}}^{+}=\text{0}$, expected as no competition for resources is possible given that the limiting resource is the metabolite essential to species 1. The interactions for species 2 follow from Eqs. (1)-(6) above, yielding the quartet. Below, we demonstrate the application of the method to a synthetic community of two auxotrophs reported previously [22].

### Application to experimental data on a community of 2 auxotrophs

We considered the experiment of Hoek *et al.* [22] on a 2-species community of auxotrophs, one (denoted $S_{W}$) incapable of synthesizing tryptophan but over-producing leucine and the other (denoted $S_{L}$) vice versa. The species were cultured alone or together in a serial passage set-up, where during each passage a 10-fold dilution was used to transfer the community population, and the two amino acids were replenished along with other nutrients. (See Results for further details.) To apply our method, we considered two limiting scenarios. In the first case, we assumed that the supplied amino acids were the limiting resource. Because the amino acids were not shared by the two species, competition for resources would now not exist. The entire supply of amino acids would effectively be ‘left-over’ for the other species. Note that the 1:8 ratio of the supply was designed based on the requirements of the auxotrophs for comparable growth. Thus, if 2 µM of tryptophan were used by $S_{W}$, incapable of synthesizing tryptophan, the entire supply of 16 µM of leucine, or the equivalent of 2 µM of tryptophan, would be available to $S_{L}$, incapable of synthesizing leucine. We therefore set the left-over resource to equal the supplied resource; *i.e.,* $\Delta R_{\text{1}}^{c}=\Delta R_{\text{2}}^{c}=R_{c}$. The rest of the calculations follow Eqs. (1)-(6). In the second case, which is likely to occur when the supplied amino acids are in excess, we assumed that some other nutrient was limiting. Because that nutrient would be shared by the two auxotrophs, we followed our method (Eqs. (1)-(6)) without modifications. The two cases thus offered bounds on the positive and negative components, which we present in Fig 5.

## Supporting information

S1 Text Estimates of the quartets using SteadyCom. (DOCX)

S2 Text Community optimization problems solved by MICOM and SteadyCom. (DOCX)

S1 Fig Estimation of the quartets for species pairs from a representative oral microbiome using SteadyCom. The distribution of (A) the net interactions and (B) their components between all pairs of 8 species in a representative human oral microbiome (see text). In (A), the net positive and net negative interactions are shown separately for clarity. (C) The individual interactions (hatched white) and their components (blue – positive; red – negative). In each panel, 1 is the species mentioned next to the row (row species) and 2 that above the column (column species) to which the panel belongs. The x-axis labels (1 or 2) indicate the species on which the influence of the other (2 or 1) is estimated. In (A) and (B), boxes show median and interquartile ranges and whiskers show extremes.
(DOCX)

S2 Fig Comparison of quartets estimated by MICOM and SteadyCom. Each dot represents a specific quartet component from the quartets estimated for the 28 species pairs of the oral microbial community we studied, obtained using MICOM (Fig 3) and SteadyCom (S1 Fig). The axes are in units of growth rates (h^-1^). The two methods show close agreement (R^2^ = 0.75).
(DOCX)

S3 Fig Variation of the quartets for select species pairs of the oral microbiome with resource supply rate. Data as in Fig 4 is presented for 9 species pairs indicated above each panel. Solid lines indicate the influence of species 2 (listed second) on species 1 (listed first) and dashed lines of species 1 on 2. Positive components are in blue and negative in red. The insets show the corresponding variations of the net interactions with the resource supply rate. Interaction strengths have units of growth rates (h^-1^).
(DOCX)

S1 Table Growth rates of species in monocultures. Units are *h*^-1^.
(DOCX)

S2 Table Growth rates of species in pairwise cocultures calculated using MICOM. Subscript 1 refers to the species in the row and 2 in the column. Units are *h*^-1^.
(DOCX)

S3 Table Net interactions between species estimated based on growth rates calculated using MICOM. Subscript 1 refers to the species in the row and 2 in the column. Units are *h*^-1^.
(DOCX)

S4 Table The quartets of the interactions between species estimated using MICOM. Subscript 1 refers to the species in the row and 2 in the column. Units are *h*^-1^.
(DOCX)

S5 Table Growth rates of species in pairwise cocultures calculated using SteadyCom. Subscript 1 refers to the species in the row and 2 in the column. Units are *h*^-1^.
(DOCX)

S6 Table Net interactions between species estimated based on growth rates calculated using SteadyCom. Subscript 1 refers to the species in the row and 2 in the column. Units are *h*^-1^.
(DOCX)

S7 Table The quartets of the interactions between species estimated using SteadyCom. Subscript 1 refers to the species in the row and 2 in the column. Units are *h*^-1^.
(DOCX)

S8 Table List of nutrients in the Western diet. The table also includes the exchange reactions the nutrients are involved in and their uptake rates (in units of millimoles/g dry cell weight/h). A ‘1’ below the species name indicates that the species uses the resource.
(DOCX)

S9 Table List of cross-fed metabolites. The cross-fed metabolites for all the species pairs of the oral microbial community we studied are listed.
(DOCX)
