# Supplementary material for: Quartet: Disentangling positive and negative components of microbial interactions
Source: PLoS Comput Biol. 2026 Jul 10;22(7):e1014502. doi: 10.1371/journal.pcbi.1014502 (PMC13384405; doi:10.1371/journal.pcbi.1014502)
Supplement: S1 Text — (DOCX) [file pcbi.1014502.s001.docx]

**S1 Text. Estimates of the quartets using SteadyCom**

Here, we summarize our findings of the estimation of the quartets using SteadyCom for community modelling. We recall that with MICOM, among the 28 species pairs we examined, resulting in 56 net interactions, 24 interactions were net positive and 32 interactions were net negative (main text, Fig 3). Further, of the 28 species pairs, 14 pairs showed exploitation or parasitic interactions, while 9 exhibited competitive interactions and 5 pairs exhibited mutualism. Using SteadyCom, we found 34 net positive (median $0.3 h^{-1}$; IQR: $[0.154, 0.57] h^{-1}$) and 22 net negative interactions (median $-0.2 h^{-1}$; IQR: $[-0.086, -0.674] h^{-1}$), with 20 pairs exhibiting exploitative interactions, 1 competitive interactions, and 7 mutualistic interactions (S1 Fig). Thus, the overall predominance of exploitative interactions and the low prevalence of mutualistic interactions was consistent with the findings with MICOM. Further, of the 112 components (constituting the 56 net interactions), we had all components to be non-zero with MICOM. With SteadyCom, we found that 106 components were non-zero. The median positive component was $0.557 h^{-1}$ (IQR: $[0.116, 0.765] h^{-1}$) and the median negative component was $-0.464 h^{-1}$ (IQR: $[-0.084, -0.691] h^{-1}$) (Fig. S1). The latter were comparable to the estimates from MICOM (main text, Fig 3).
