# Supplementary material for: Quartet: Disentangling positive and negative components of microbial interactions
Source: PLoS Comput Biol. 2026 Jul 10;22(7):e1014502. doi: 10.1371/journal.pcbi.1014502 (PMC13384405; doi:10.1371/journal.pcbi.1014502)
Supplement: S2 Text — (DOCX) [file pcbi.1014502.s002.docx]

**S2 Text. Community optimization problems solved by MICOM and SteadyCom**

Here, we briefly outline the optimization problems solved by MICOM and SteadyCom to predict the species growth rates in a community environment.

**MICOM:**

The community growth rate, $\mu_{c}$, is maximized. Thus, the optimization problem is

$$\mathrm{maximize} \mu_{c}= \sum_{i} \alpha_{i}\mu_{i}$$

subject to

$$\forall i:S_{i}v_{i}=0$$

$$\mu_{i}= v_{i, biomass}\geq\mu_{i}^{min}$$

$$l_{i} \leq v_{i} \leq u_{i}$$

where $\alpha_{i}$ is the biomass abundance of species $i$; $\mu_{i}$ is its growth rate; $v_{i, biomass}$ is its biomass flux; $\mu_{i}^{min}$ is a specified minimum growth rate; $l_{i}$and $u_{i}$ are lower and upper bounds on the fluxes $v_{i}$; and $S_{i}$ is the stoichiometric matrix of species $i$. The constraints on the fluxes also apply to exchanges with the environment. The constraints, termed community constraints, have been applied as specified (see [32] in the main text). MICOM relaxes the balanced growth condition and solves for the $\mu_{i}$ by solving the quadratic minimization problem

$$\mathrm{minimize} \sum_{i} \mu_{i}^{2}$$

subject to

$$\mu_{c}\geq\propto\mu_{c}^{max}$$

where $\mu_{c}^{max}$ is the solution to the maximization problem above and $\propto$ is a specified constant reflecting the trade-off that lets the community growth rate deviate from the maximum possible growth rate while allowing for interspecies variations in growth rates.

**SteadyCom:**

The community growth rate, $\mu_{c}$, is maximized. Thus, the optimization problem is

$$\mathrm{maximize} \mu_{c}$$

subject to

$$\forall i:S_{i}v_{i}=0$$

$$v_{i, biomass}=\mu_{C}x_{i}$$

$$l_{i} \leq v_{i} \leq u_{i}$$

$$x_{i}\geq0$$

$$\sum_{i} x_{i}=1$$

The solution is thus subject to the same community constraints as above. Further, the fluxes $v_{i}$ are now weighted by the relative abundances, $x_{i}$, of the respective species in balancing their exchanges with the environment.

The implementation was performed using the packages available in Python and MATLAB, respectively (see Methods).
