# Supplementary figures and images for: Quartet: Disentangling positive and negative components of microbial interactions
Source: PLoS Comput Biol. 2026 Jul 10;22(7):e1014502. doi: 10.1371/journal.pcbi.1014502 (PMC13384405; doi:10.1371/journal.pcbi.1014502)

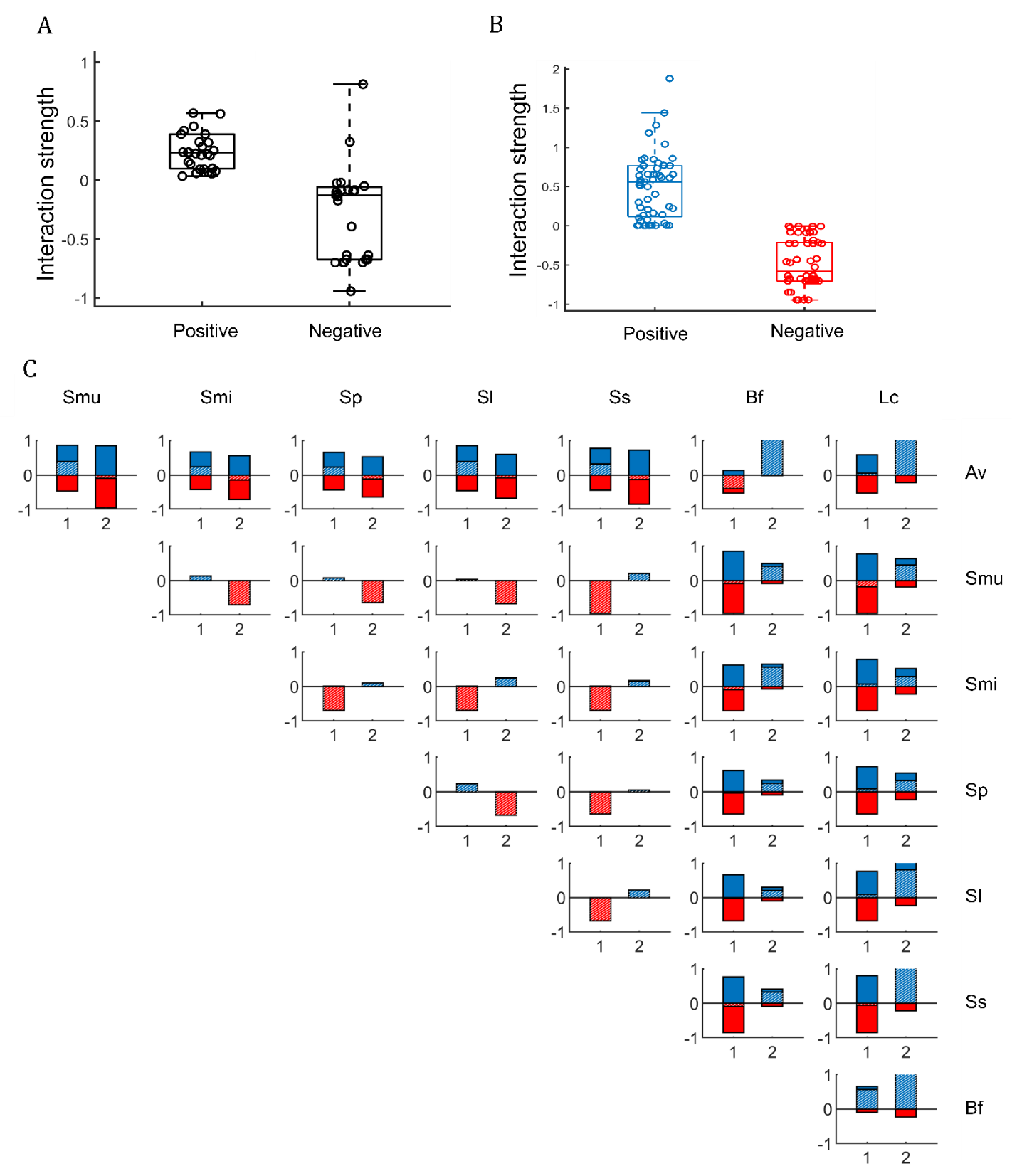

Supplement: S1 Fig — The distribution of (A) the net interactions and (B) their components between all pairs of 8 species in a representative human oral microbiome (see text). In (A), the net positive and net negative interactions are shown separately for clarity. (C) The individual interactions (hatched white) and their components (blue – positive; red – negative). In each panel, 1 is the species mentioned next to the row (row species) and 2 that above the column (column species) to which the panel belongs. The x-axis labels (1 or 2) indicate the species on which the influence of the other (2 or 1) is estimated. In (A) and (B), boxes show median and interquartile ranges and whiskers show extremes. (DOCX) [file pcbi.1014502.s003.docx]

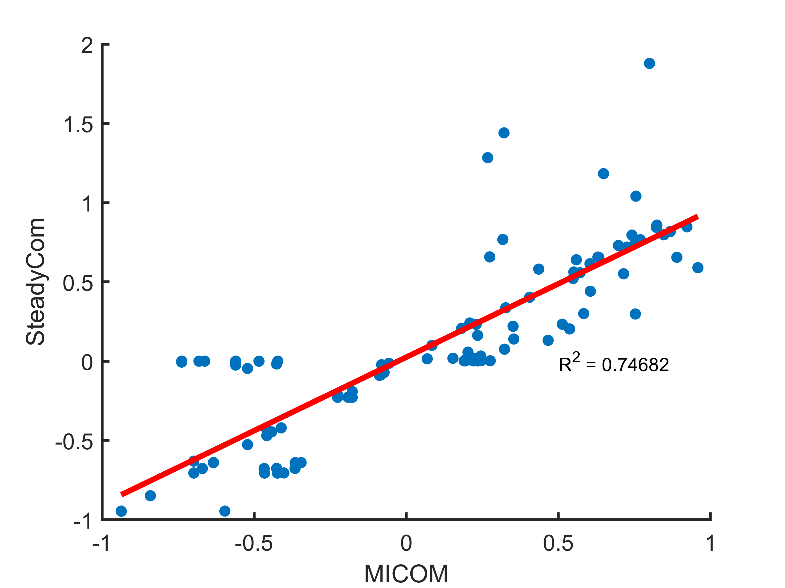

Supplement: S2 Fig — Each dot represents a specific quartet component from the quartets estimated for the 28 species pairs of the oral microbial community we studied, obtained using MICOM (Fig 3) and SteadyCom (S1 Fig). The axes are in units of growth rates (h-1). The two methods show close agreement (R2 = 0.75). (DOCX) [file pcbi.1014502.s004.docx]

Smu-Smi

Av-Smi

Av-Smu


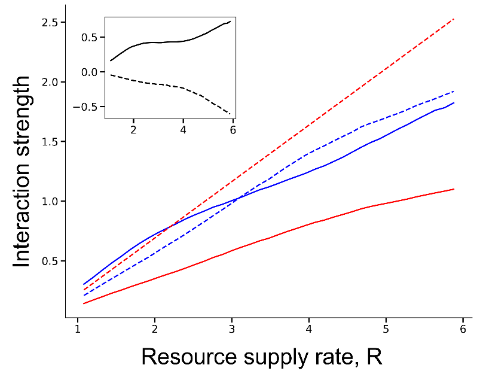

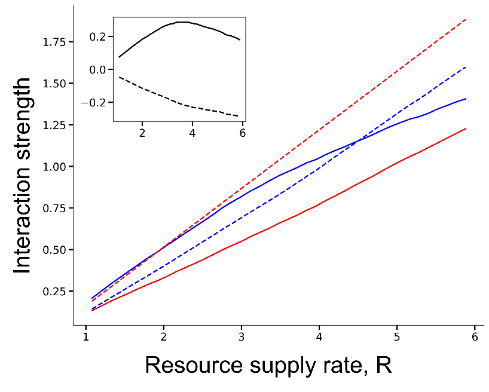

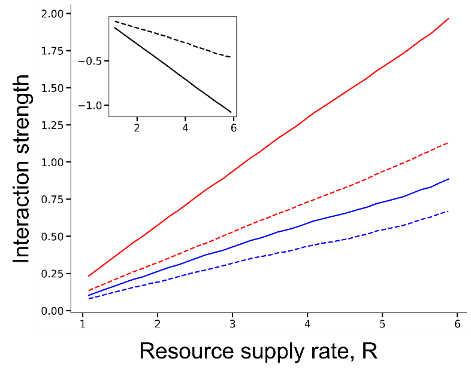


Smi-Ss

Smu-Lc

Smi-Sl


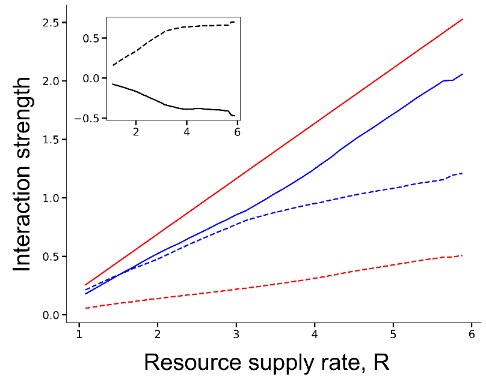

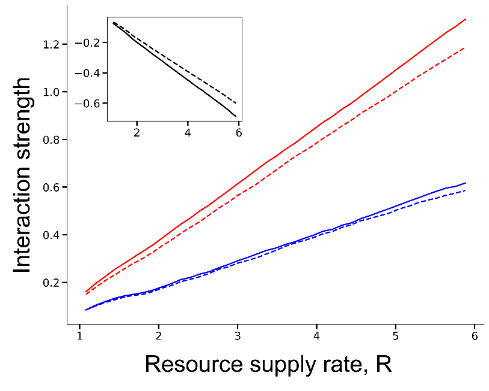

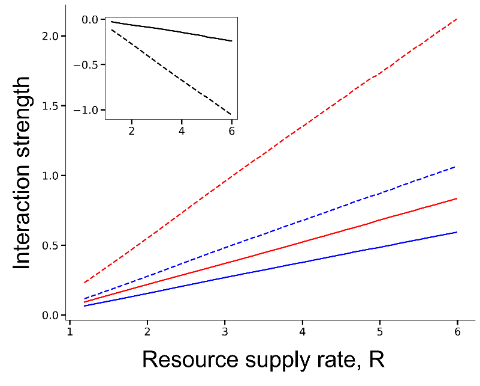


Sl-Ss

Sp-Lc

Sp-Bf


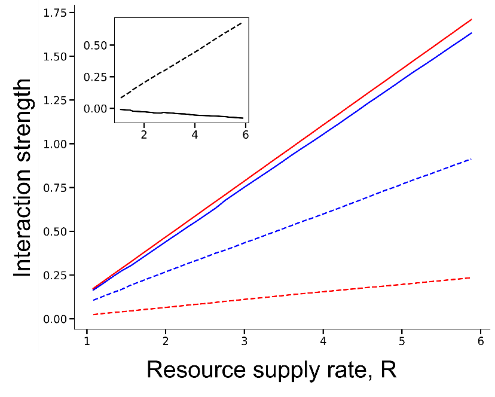

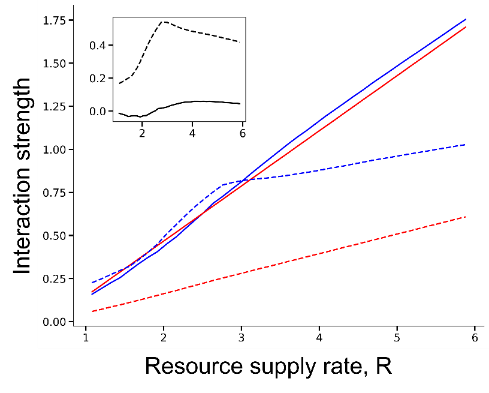

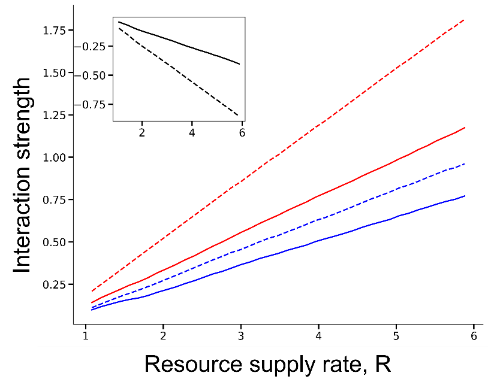

Supplement: S3 Fig — Data as in Fig 4 is presented for 9 species pairs indicated above each panel. Solid lines indicate the influence of species 2 (listed second) on species 1 (listed first) and dashed lines of species 1 on 2. Positive components are in blue and negative in red. The insets show the corresponding variations of the net interactions with the resource supply rate. Interaction strengths have units of growth rates (h-1). (DOCX) [file pcbi.1014502.s005.docx]
