# Supplementary material for: Quartet: Disentangling positive and negative components of microbial interactions
Source: PLoS Comput Biol. 2026 Jul 10;22(7):e1014502. doi: 10.1371/journal.pcbi.1014502 (PMC13384405; doi:10.1371/journal.pcbi.1014502)
Supplement: S1 Table — Units are h-1. (DOCX) [file pcbi.1014502.s006.docx]

| **Av** | **Smu** | **Smi** | **Sp** | **Sl** | **Ss** | **Bf** | **Lc** |
| --- | --- | --- | --- | --- | --- | --- | --- |
| 0.522 | 0.937 | 0.698 | 0.634 | 0.670 | 0.841 | 0.089 | 0.226 |
