# Supplementary material for: Quartet: Disentangling positive and negative components of microbial interactions
Source: PLoS Comput Biol. 2026 Jul 10;22(7):e1014502. doi: 10.1371/journal.pcbi.1014502 (PMC13384405; doi:10.1371/journal.pcbi.1014502)
Supplement: S2 Table — Subscript 1 refers to the species in the row and 2 in the column. Units are h-1. (DOCX) [file pcbi.1014502.s007.docx]

|  | **Smu** | | **Smi** | | **Sp** | | **Sl** | | **Ss** | | **Bf** | | **Lc** | |
| --- | --- | --- | --- | --- | --- | --- | --- | --- | --- | --- | --- | --- | --- | --- |
|  | $\mu_{1}^{c}$ | $\mu_{2}^{c}$ | $\mu_{1}^{c}$ | $\mu_{2}^{c}$ | $\mu_{1}^{c}$ | $\mu_{2}^{c}$ | $\mu_{1}^{c}$ | $\mu_{2}^{c}$ | $\mu_{1}^{c}$ | $\mu_{2}^{c}$ | $\mu_{1}^{c}$ | $\mu_{2}^{c}$ | $\mu_{1}^{c}$ | $\mu_{2}^{c}$ |
| **Av** | 0.887 | 0.822 | 0.741 | 0.552 | 0.696 | 0.549 | 0.923 | 0.959 | 0.832 | 0.725 | 0.466 | 0.657 | 0.436 | 0.356 |
| **Smu** |  |  | 0.552 | 0.543 | 0.522 | 0.509 | 0.501 | 0.495 | 0.535 | 0.539 | 0.869 | 0.519 | 0.742 | 0.653 |
| **Smi** |  |  |  |  | 0.070 | 0.661 | 0.454 | 0.453 | 0.475 | 0.515 | 0.572 | 0.600 | 0.559 | 0.585 |
| **Sp** |  |  |  |  |  |  | 0.441 | 0.438 | 0.422 | 0.483 | 0.604 | 0.333 | 0.698 | 0.714 |
| **Sl** |  |  |  |  |  |  |  |  | 0.520 | 0.530 | 0.890 | 0.753 | 0.318 | 0.755 |
| **Ss** |  |  |  |  |  |  |  |  |  |  | 0.769 | 0.414 | 0.847 | 0.800 |
| **Bf** |  |  |  |  |  |  |  |  |  |  |  |  | 0.275 | 0.268 |
