# Supplementary material for: Quartet: Disentangling positive and negative components of microbial interactions
Source: PLoS Comput Biol. 2026 Jul 10;22(7):e1014502. doi: 10.1371/journal.pcbi.1014502 (PMC13384405; doi:10.1371/journal.pcbi.1014502)
Supplement: S3 Table — Subscript 1 refers to the species in the row and 2 in the column. Units are h-1. (DOCX) [file pcbi.1014502.s008.docx]

|  | **Smu** | | **Smi** | | **Sp** | | **Sl** | | **Ss** | | **Bf** | | **Lc** | |
| --- | --- | --- | --- | --- | --- | --- | --- | --- | --- | --- | --- | --- | --- | --- |
|  | $\Delta_{1\leftarrow2}^{net}$ | $\Delta_{2\leftarrow1}^{net}$ | $\Delta_{1\leftarrow2}^{net}$ | $\Delta_{2\leftarrow1}^{net}$ | $\Delta_{1\leftarrow2}^{net}$ | $\Delta_{2\leftarrow1}^{net}$ | $\Delta_{1\leftarrow2}^{net}$ | $\Delta_{2\leftarrow1}^{net}$ | $\Delta_{1\leftarrow2}^{net}$ | $\Delta_{2\leftarrow1}^{net}$ | $\Delta_{1\leftarrow2}^{net}$ | $\Delta_{2\leftarrow1}^{net}$ | $\Delta_{1\leftarrow2}^{net}$ | $\Delta_{2\leftarrow1}^{net}$ |
| **Av** | 0.365 | -0.114 | 0.220 | -0.146 | 0.174 | -0.085 | 0.401 | 0.289 | 0.310 | -0.116 | -0.055 | 0.568 | -0.086 | 0.130 |
| **Smu** |  |  | -0.384 | -0.155 | -0.415 | -0.125 | -0.436 | -0.175 | -0.402 | -0.302 | -0.068 | 0.430 | -0.195 | 0.428 |
| **Smi** |  |  |  |  | -0.628 | 0.027 | -0.245 | -0.217 | -0.223 | -0.325 | -0.127 | 0.511 | -0.139 | 0.359 |
| **Sp** |  |  |  |  |  |  | -0.193 | -0.232 | -0.211 | -0.358 | -0.029 | 0.244 | 0.064 | 0.489 |
| **Sl** |  |  |  |  |  |  |  |  | -0.150 | -0.311 | 0.220 | 0.664 | -0.353 | 0.530 |
| **Ss** |  |  |  |  |  |  |  |  |  |  | -0.071 | 0.325 | 0.006 | 0.574 |
| **Bf** |  |  |  |  |  |  |  |  |  |  |  |  | 0.186 | 0.042 |
