# Supplementary material for: Quartet: Disentangling positive and negative components of microbial interactions
Source: PLoS Comput Biol. 2026 Jul 10;22(7):e1014502. doi: 10.1371/journal.pcbi.1014502 (PMC13384405; doi:10.1371/journal.pcbi.1014502)
Supplement: S5 Table — Subscript 1 refers to the species in the row and 2 in the column. Units are h-1. (DOCX) [file pcbi.1014502.s010.docx]

|  | **Smu** | | **Smi** | | **Sp** | | **Sl** | | **Ss** | | **Bf** | | **Lc** | |
| --- | --- | --- | --- | --- | --- | --- | --- | --- | --- | --- | --- | --- | --- | --- |
|  | $\mu_{1}^{c}$ | $\mu_{2}^{c}$ | $\mu_{1}^{c}$ | $\mu_{2}^{c}$ | $\mu_{1}^{c}$ | $\mu_{2}^{c}$ | $\mu_{1}^{c}$ | $\mu_{2}^{c}$ | $\mu_{1}^{c}$ | $\mu_{2}^{c}$ | $\mu_{1}^{c}$ | $\mu_{2}^{c}$ | $\mu_{1}^{c}$ | $\mu_{2}^{c}$ |
| **Av** | 0.915 | 0.844 | 0.763 | 0.563 | 0.751 | 0.522 | 0.915 | 0.590 | 0.844 | 0.720 | 0.132 | 1.250 | 0.581 | 1.440 |
| **Smu** |  |  | 1.080 | 0.004 | 1.021 | 0.002 | 0.978 | 0.002 | 0.003 | 1.056 | 0.860 | 0.508 | 0.770 | 0.684 |
| **Smi** |  |  |  |  | 0.004 | 0.737 | 0.006 | 0.913 | 0.006 | 1.003 | 0.611 | 0.652 | 0.771 | 0.513 |
| **Sp** |  |  |  |  |  |  | 0.872 | 0.002 | 0.002 | 0.906 | 0.615 | 0.340 | 0.730 | 0.551 |
| **Sl** |  |  |  |  |  |  |  |  | 0.003 | 1.069 | 0.655 | 0.300 | 0.768 | 1.041 |
| **Ss** |  |  |  |  |  |  |  |  |  |  | 0.766 | 0.412 | 0.797 | 1.890 |
| **Bf** |  |  |  |  |  |  |  |  |  |  |  |  | 0.658 | 1.283 |
