# Supplementary material for: Quartet: Disentangling positive and negative components of microbial interactions
Source: PLoS Comput Biol. 2026 Jul 10;22(7):e1014502. doi: 10.1371/journal.pcbi.1014502 (PMC13384405; doi:10.1371/journal.pcbi.1014502)
Supplement: S6 Table — Subscript 1 refers to the species in the row and 2 in the column. Units are h-1. (DOCX) [file pcbi.1014502.s011.docx]

|  | **Smu** | | **Smi** | | **Sp** | | **Sl** | | **Ss** | | **Bf** | | **Lc** | |
| --- | --- | --- | --- | --- | --- | --- | --- | --- | --- | --- | --- | --- | --- | --- |
|  | $\Delta_{1\leftarrow2}^{net}$ | $\Delta_{2\leftarrow1}^{net}$ | $\Delta_{1\leftarrow2}^{net}$ | $\Delta_{2\leftarrow1}^{net}$ | $\Delta_{1\leftarrow2}^{net}$ | $\Delta_{2\leftarrow1}^{net}$ | $\Delta_{1\leftarrow2}^{net}$ | $\Delta_{2\leftarrow1}^{net}$ | $\Delta_{1\leftarrow2}^{net}$ | $\Delta_{2\leftarrow1}^{net}$ | $\Delta_{1\leftarrow2}^{net}$ | $\Delta_{2\leftarrow1}^{net}$ | $\Delta_{1\leftarrow2}^{net}$ | $\Delta_{2\leftarrow1}^{net}$ |
| **Av** | 0.389 | -0.102 | 0.236 | -0.142 | 0.225 | -0.118 | 0.389 | -0.087 | 0.317 | -0.129 | -0.395 | 1.161 | 0.054 | 1.212 |
| **Smu** |  |  | 0.134 | -0.701 | 0.076 | -0.637 | 0.033 | -0.674 | -0.943 | 0.208 | -0.068 | 0.430 | -0.195 | 0.428 |
| **Smi** |  |  |  |  | -0.700 | 0.098 | -0.699 | 0.237 | -0.699 | 0.154 | -0.094 | 0.562 | 0.066 | 0.285 |
| **Sp** |  |  |  |  |  |  | 0.232 | -0.674 | -0.638 | 0.057 | -0.025 | 0.250 | 0.090 | 0.324 |
| **Sl** |  |  |  |  |  |  |  |  | -0.674 | 0.220 | -0.021 | 0.211 | 0.092 | 0.813 |
| **Ss** |  |  |  |  |  |  |  |  |  |  | -0.083 | 0.322 | -0.051 | 1.664 |
| **Bf** |  |  |  |  |  |  |  |  |  |  |  |  | 0.568 | 1.056 |
