# Supplementary material for: Quartet: Disentangling positive and negative components of microbial interactions
Source: PLoS Comput Biol. 2026 Jul 10;22(7):e1014502. doi: 10.1371/journal.pcbi.1014502 (PMC13384405; doi:10.1371/journal.pcbi.1014502)
Supplement: S8 Table — The table also includes the exchange reactions the nutrients are involved in and their uptake rates (in units of millimoles/g dry cell weight/h). A ‘1’ below the species name indicates that the species uses the resource. (DOCX) [file pcbi.1014502.s013.docx]

|  | | | | | | | | | | | |
| --- | --- | --- | --- | --- | --- | --- | --- | --- | --- | --- | --- |
|  |  |  |  |  |  |  |  |  |  |  |  |
| Nutreints | Exchange reaction ID | Metabolite name | Uptake rate | Av | Smu | Smi | Sp | Sl | Ss | Bf | Lc |
| arab_L | EX_arab_L(e) | L-arabinose | 0.17878295 | 1 | 1 | 1 | 1 | 1 | 1 | 1 | 1 |
| cellb | EX_cellb(e) | Cellobiose | 0.07449289 | 0 | 1 | 0 | 0 | 1 | 1 | 0 | 1 |
| drib | EX_drib(e) | 2-deoxy-D-ribose | 0.17878295 | 1 | 0 | 1 | 0 | 0 | 0 | 0 | 1 |
| fru | EX_fru(e) | D-Fructose | 0.14898579 | 1 | 1 | 1 | 1 | 1 | 1 | 0 | 1 |
| fuc_L | EX_fuc_L(e) | L-fucose | 0.14898579 | 0 | 0 | 0 | 0 | 0 | 0 | 0 | 0 |
| gal | EX_gal(e) | D-Galactose | 0.14898579 | 0 | 1 | 1 | 1 | 0 | 1 | 0 | 1 |
| glc_D | EX_glc(e) | D-glucose | 0.14898579 | 0 | 0 | 0 | 0 | 0 | 0 | 0 | 0 |
| glcn | EX_glcn(e) | D-gluconate | 0.14898579 | 0 | 0 | 0 | 0 | 0 | 0 | 0 | 1 |
| lcts | EX_lcts(e) | Lactose | 0.07449289 | 1 | 1 | 1 | 1 | 0 | 1 | 1 | 1 |
| malt | EX_malt(e) | Maltose | 0.07449289 | 1 | 1 | 1 | 1 | 1 | 1 | 1 | 1 |
| man | EX_man(e) | D-Mannose | 0.14898579 | 1 | 1 | 1 | 1 | 1 | 1 | 0 | 1 |
| melib | EX_melib(e) | Melibiose | 0.07449289 | 0 | 0 | 0 | 0 | 0 | 0 | 1 | 1 |
| mnl | EX_mnl(e) | D-Mannitol | 0.14898579 | 1 | 1 | 1 | 1 | 1 | 1 | 0 | 1 |
| oxa | EX_oxa(e) | Oxalate(2-) | 0.44695737 | 0 | 0 | 0 | 0 | 0 | 0 | 0 | 0 |
| rib_D | EX_rib_D(e) | D-ribose | 0.17878295 | 1 | 0 | 1 | 0 | 0 | 0 | 1 | 1 |
| rmn | EX_rmn(e) | L-Rhamnose | 0.14898579 | 0 | 0 | 0 | 0 | 0 | 0 | 0 | 0 |
| sucr | EX_sucr(e) | Sucrose | 0.07449289 | 1 | 1 | 1 | 1 | 1 | 1 | 1 | 1 |
| tre | EX_tre(e) | Trehalose | 0.07449289 | 1 | 1 | 1 | 1 | 1 | 1 | 0 | 1 |
| xyl_D | EX_xyl_D(e) | D-xylose | 0.17878295 | 0 | 0 | 0 | 0 | 0 | 0 | 1 | 1 |
| strch1 | EX_strch1(e) | Starch | 0.25733909 | 0 | 1 | 0 | 0 | 1 | 0 | 0 | 0 |
| amylopect900 | EX_amylopect900(e) | Amylopectin | 0.00001567 | 0 | 0 | 0 | 0 | 0 | 0 | 0 | 0 |
| amylose300 | EX_amylose300(e) | Amylose | 0.00004702 | 0 | 0 | 0 | 0 | 0 | 0 | 0 | 0 |
| arabinan101 | EX_arabinan101(e) | Arabinan | 0.00016628 | 0 | 0 | 0 | 0 | 0 | 0 | 0 | 0 |
| arabinogal | EX_arabinogal(e) | Larch arabinogalactan | 0.00002191 | 0 | 0 | 0 | 0 | 0 | 0 | 0 | 0 |
| arabinoxyl | EX_arabinoxyl(e) | Arabinoxylan | 0.00030665 | 0 | 0 | 0 | 0 | 0 | 0 | 0 | 0 |
| bglc | EX_bglc(e) | Beta-glucan | 0.00000007 | 0 | 0 | 0 | 0 | 0 | 0 | 0 | 0 |
| cellul | EX_cellul(e) | Cellulose | 0.00002821 | 0 | 0 | 0 | 0 | 0 | 0 | 0 | 0 |
| dextran40 | EX_dextran40(e) | Dextran 40, 1,6-alpha-Dglucan | 0.00017632 | 0 | 0 | 0 | 0 | 0 | 0 | 0 | 0 |
| galmannan | EX_galmannan(e) | Carob galactomannan | 0.00001411 | 0 | 0 | 0 | 0 | 0 | 0 | 0 | 0 |
| glcmannan | EX_glcmannan(e) | Konjac glucomannan | 0.00003288 | 0 | 0 | 0 | 0 | 0 | 0 | 0 | 0 |
| homogal | EX_homogal(e) | Homogalacturonan | 0.00012823 | 0 | 0 | 0 | 0 | 0 | 0 | 0 | 0 |
| inulin | EX_inulin(e) | Chicory inulin | 0.00047019 | 0 | 1 | 0 | 0 | 0 | 0 | 0 | 0 |
| kestopt | EX_kestopt(e) | Kestopentaose | 0.00282117 | 0 | 1 | 1 | 0 | 0 | 0 | 0 | 1 |
| levan1000 | EX_levan1000(e) | Levan, 1000 fructose units | 0.00001411 | 0 | 0 | 0 | 0 | 0 | 0 | 0 | 0 |
| lichn | EX_lichn(e) | Lichenin from Icelandic moss | 0.00008298 | 0 | 0 | 0 | 0 | 0 | 0 | 0 | 0 |
| lmn30 | EX_lmn30(e) | Laminarin | 0.00047019 | 0 | 0 | 0 | 0 | 0 | 0 | 0 | 0 |
| pect | EX_pect(e) | Pectin | 0.00003339 | 0 | 0 | 0 | 0 | 0 | 0 | 0 | 0 |
| pullulan1200 | EX_pullulan1200(e) | Pullulan | 0.00001175 | 0 | 0 | 0 | 0 | 1 | 0 | 0 | 0 |
| raffin | EX_raffin(e) | Raffinose | 0.00470194 | 1 | 1 | 0 | 0 | 0 | 0 | 1 | 0 |
| rhamnogalurI | EX_rhamnogalurI(e) | Potato rhamnogalacturonan I | 0.00001449 | 0 | 0 | 0 | 0 | 0 | 0 | 0 | 0 |
| rhamnogalurII | EX_rhamnogalurII(e) | Wine rhamnogalacturonan II | 0.00026699 | 0 | 0 | 0 | 0 | 0 | 0 | 0 | 0 |
| starch1200 | EX_starch1200(e) | Resistant starch | 0.00001175 | 0 | 0 | 0 | 0 | 0 | 0 | 0 | 0 |
| xylan | EX_xylan(e) | Oat spelt xylan | 0.00003206 | 0 | 0 | 0 | 0 | 0 | 0 | 0 | 0 |
| xyluglc | EX_xyluglc(e) | Xyluglucan | 0.00001315 | 0 | 0 | 0 | 0 | 0 | 0 | 0 | 0 |
| arachd | EX_arachd(e) | Arachidonate | 0.00332813 | 0 | 0 | 0 | 0 | 0 | 0 | 0 | 0 |
| chsterol | EX_chsterol(e) | Cholesterol | 0.00495795 | 0 | 0 | 0 | 0 | 0 | 0 | 0 | 1 |
| glyc | EX_glyc(e) | Glycerol | 1.79965486 | 1 | 1 | 1 | 1 | 1 | 1 | 1 | 1 |
| hdca | EX_hdca(e) | Hexadecanoate (n-C16:0) | 0.3963709 | 0 | 0 | 0 | 0 | 0 | 0 | 0 | 0 |
| hdcea | EX_hdcea(e) | Hexadecenoate (n-C16:1) | 0.03651697 | 0 | 0 | 0 | 0 | 0 | 0 | 0 | 0 |
| lnlc | EX_lnlc(e) | Linoleate | 0.35910921 | 0 | 0 | 0 | 0 | 0 | 0 | 0 | 0 |
| lnlnca | EX_lnlnca(e) | Alpha-linolenate | 0.01756512 | 0 | 0 | 0 | 0 | 0 | 0 | 0 | 0 |
| lnlncg | EX_lnlncg(e) | Gamma-linolenate | 0.01756512 | 0 | 0 | 0 | 0 | 0 | 0 | 0 | 0 |
| ocdca | EX_ocdca(e) | Octadecanoate (n-C18:0) | 0.1692826 | 1 | 1 | 0 | 0 | 0 | 1 | 0 | 0 |
| ocdcea | EX_ocdcea(e) | Octadecenoate (n-C18:1) | 0.68144465 | 0 | 0 | 0 | 0 | 0 | 0 | 0 | 0 |
| octa | EX_octa(e) | Octanoate (n-C8:0) | 0.01294272 | 0 | 0 | 0 | 0 | 0 | 0 | 0 | 0 |
| ttdca | EX_ttdca(e) | Tetradecanoate (n-C14:0) | 0.06867567 | 1 | 0 | 0 | 0 | 0 | 0 | 0 | 0 |
| ala_L | EX_ala_L(e) | L-alanine | 1 | 1 | 1 | 1 | 1 | 1 | 1 | 1 | 1 |
| arg_L | EX_arg_L(e) | L-arginine | 0.15 | 1 | 1 | 1 | 1 | 0 | 1 | 1 | 1 |
| asn_L | EX_asn_L(e) | L-asparagine | 0.225 | 1 | 1 | 1 | 1 | 1 | 1 | 1 | 1 |
| asp_L | EX_asp_L(e) | L-aspartate | 0.225 | 1 | 1 | 1 | 1 | 1 | 1 | 1 | 1 |
| cys_L | EX_cys_L(e) | L-cysteine | 1 | 1 | 1 | 1 | 1 | 1 | 1 | 1 | 1 |
| gln_L | EX_gln_L(e) | L-glutamine | 0.18 | 1 | 1 | 1 | 1 | 1 | 1 | 1 | 1 |
| glu_L | EX_glu_L(e) | L-glutamate | 0.18 | 1 | 1 | 1 | 1 | 1 | 1 | 1 | 1 |
| gly | EX_gly(e) | Glycine | 0.45 | 1 | 1 | 1 | 1 | 1 | 1 | 1 | 1 |
| his_L | EX_his_L(e) | L-histidine | 0.15 | 1 | 1 | 1 | 1 | 1 | 1 | 1 | 1 |
| ile_L | EX_ile_L(e) | L-isoleucine | 0.15 | 1 | 1 | 1 | 1 | 1 | 1 | 1 | 1 |
| leu_L | EX_leu_L(e) | L-leucine | 0.15 | 1 | 1 | 1 | 1 | 1 | 1 | 1 | 1 |
| lys_L | EX_lys_L(e) | L-lysine | 0.15 | 1 | 1 | 1 | 1 | 1 | 1 | 1 | 1 |
| met_L | EX_met_L(e) | L-methionine | 0.18 | 1 | 1 | 1 | 1 | 1 | 1 | 1 | 1 |
| phe_L | EX_phe_L(e) | L-phenylalanine | 1 | 1 | 1 | 1 | 1 | 1 | 1 | 1 | 1 |
| pro_L | EX_pro_L(e) | L-proline | 0.18 | 1 | 1 | 1 | 1 | 1 | 1 | 1 | 1 |
| ser_L | EX_ser_L(e) | L-serine | 1 | 1 | 1 | 1 | 1 | 1 | 1 | 1 | 1 |
| thr_L | EX_thr_L(e) | L-threonine | 0.225 | 1 | 1 | 1 | 1 | 1 | 1 | 1 | 1 |
| trp_L | EX_trp_L(e) | L-tryptophan | 0.08181818 | 0 | 0 | 0 | 0 | 0 | 0 | 1 | 1 |
| tyr_L | EX_tyr_L(e) | L-tyrosine | 1 | 1 | 1 | 1 | 1 | 1 | 1 | 1 | 1 |
| val_L | EX_val_L(e) | L-valine | 0.18 | 1 | 1 | 1 | 1 | 1 | 1 | 1 | 1 |
| 12dgr180 | EX_12dgr180(e) | 1,2-Diacyl-sn-glycerol (dioctadecanoyl, n-C18:0) | 1 | 0 | 0 | 0 | 0 | 0 | 1 | 0 | 0 |
| 26dap_M | EX_26dap_M(e) | meso-2,6-Diaminoheptanedioate | 1 | 1 | 1 | 1 | 1 | 1 | 1 | 0 | 0 |
| 2dmmq8 | EX_2dmmq8(e) | 2-Demethylmenaquinone 8 | 1 | 0 | 1 | 1 | 1 | 1 | 1 | 0 | 0 |
| 2obut | EX_2obut(e) | 2-Oxobutanoate | 1 | 0 | 1 | 1 | 1 | 1 | 1 | 0 | 0 |
| 3mop | EX_3mop(e) | 3-methyl-2-oxopentanoate | 1 | 0 | 0 | 0 | 0 | 0 | 0 | 0 | 0 |
| 4abz | EX_4abz(e) | 4-Aminobenzoate | 1 | 1 | 0 | 0 | 0 | 0 | 0 | 0 | 0 |
| 4hbz | EX_4hbz(e) | 4-hydroxybenzoate | 1 | 1 | 1 | 0 | 0 | 1 | 0 | 0 | 0 |
| ac | EX_ac(e) | Acetate | 1 | 1 | 1 | 1 | 1 | 1 | 1 | 1 | 1 |
| acgam | EX_acgam(e) | N-acetyl-D-glucosamine | 1 | 1 | 1 | 1 | 1 | 1 | 1 | 0 | 0 |
| acmana | EX_acmana(e) | N-acetyl-D-mannosamine | 1 | 0 | 0 | 0 | 0 | 0 | 0 | 0 | 0 |
| acnam | EX_acnam(e) | N-acetylneuraminate | 1 | 0 | 0 | 0 | 0 | 0 | 1 | 0 | 0 |
| ade | EX_ade(e) | Adenine | 1 | 0 | 1 | 1 | 1 | 1 | 1 | 1 | 0 |
| adn | EX_adn(e) | Adenosine | 1 | 0 | 0 | 1 | 1 | 0 | 0 | 0 | 0 |
| adocbl | EX_adocbl(e) | Adenosylcobalamin | 1 | 0 | 0 | 0 | 0 | 0 | 0 | 0 | 0 |
| ala_D | EX_ala_D(e) | D-alanine | 1 | 1 | 0 | 0 | 0 | 0 | 0 | 0 | 1 |
| amp | EX_amp(e) | AMP | 1 | 0 | 0 | 0 | 1 | 0 | 1 | 0 | 0 |
| arab_D | EX_arab_D(e) | D-Arabinose | 1 | 0 | 0 | 0 | 0 | 0 | 0 | 0 | 0 |
| btn | EX_btn(e) | Biotin | 1 | 1 | 1 | 1 | 1 | 1 | 1 | 1 | 0 |
| ca2 | EX_ca2(e) | Calcium(2+) | 1 | 1 | 1 | 1 | 1 | 1 | 1 | 1 | 1 |
| cbl1 | EX_cbl1(e) | Cob(I)alamin | 1 | 0 | 0 | 1 | 0 | 1 | 1 | 0 | 0 |
| cgly | EX_cgly(e) | L-cysteinylglycine | 1 | 1 | 1 | 1 | 1 | 1 | 1 | 1 | 1 |
| chol | EX_chol(e) | Choline | 1 | 1 | 1 | 0 | 0 | 0 | 0 | 0 | 1 |
| chor | EX_chor(e) | Chorismate | 1 | 0 | 0 | 0 | 0 | 0 | 0 | 0 | 0 |
| cit | EX_cit(e) | Citrate | 1 | 1 | 1 | 0 | 0 | 0 | 0 | 0 | 1 |
| cl | EX_cl(e) | Chloride | 1 | 1 | 1 | 1 | 1 | 1 | 1 | 1 | 1 |
| cobalt2 | EX_cobalt2(e) | Co2+ | 1 | 1 | 1 | 1 | 1 | 1 | 1 | 1 | 1 |
| csn | EX_csn(e) | Cytosine | 1 | 1 | 1 | 1 | 1 | 1 | 1 | 0 | 0 |
| cu2 | EX_cu2(e) | Cu2+ | 1 | 1 | 1 | 1 | 1 | 1 | 1 | 1 | 1 |
| dad_2 | EX_dad_2(e) | 2-deoxyadenosine | 1 | 0 | 1 | 1 | 1 | 1 | 1 | 0 | 0 |
| dcyt | EX_dcyt(e) | Deoxycytidine | 1 | 1 | 1 | 1 | 1 | 1 | 1 | 0 | 0 |
| ddca | EX_ddca(e) | Laurate | 1 | 0 | 0 | 0 | 0 | 0 | 0 | 0 | 0 |
| dgsn | EX_dgsn(e) | Deoxyguanosine | 1 | 0 | 1 | 1 | 1 | 1 | 1 | 0 | 0 |
| fe2 | EX_fe2(e) | Fe2+ | 1 | 1 | 1 | 1 | 1 | 1 | 1 | 1 | 1 |
| fe3 | EX_fe3(e) | Fe3+ | 1 | 1 | 1 | 1 | 1 | 1 | 1 | 1 | 1 |
| fe3dcit | EX_fe3dcit(e) | Fe(III)dicitrate | 1 | 0 | 0 | 0 | 0 | 0 | 0 | 0 | 0 |
| fald | EX_fald(e) | Formaldehyde | 1 | 0 | 0 | 0 | 0 | 0 | 0 | 0 | 0 |
| fol | EX_fol(e) | Folate | 1 | 1 | 1 | 1 | 1 | 1 | 1 | 0 | 1 |
| for | EX_for(e) | Formate | 1 | 1 | 1 | 1 | 1 | 1 | 1 | 1 | 1 |
| fum | EX_fum(e) | Fumarate | 1 | 0 | 0 | 0 | 0 | 0 | 0 | 0 | 0 |
| gam | EX_gam(e) | D-Glucosamine | 1 | 1 | 1 | 1 | 1 | 1 | 1 | 0 | 0 |
| glu_D | EX_glu_D(e) | D-Glutamate | 1 | 0 | 0 | 0 | 0 | 0 | 0 | 0 | 0 |
| glyc3p | EX_glyc3p(e) | Glycerol 3-phosphate | 1 | 0 | 0 | 0 | 0 | 0 | 0 | 0 | 1 |
| gthox | EX_gthox(e) | Oxidized glutathione | 1 | 0 | 0 | 0 | 0 | 0 | 0 | 0 | 0 |
| gthrd | EX_gthrd(e) | Reduced glutathione | 1 | 1 | 0 | 0 | 0 | 0 | 0 | 0 | 0 |
| gua | EX_gua(e) | Guanine | 1 | 1 | 1 | 1 | 1 | 1 | 1 | 0 | 0 |
| h | EX_h(e) | Proton | 1 | 1 | 1 | 1 | 1 | 1 | 1 | 1 | 1 |
| H2 | EX_h2(e) | Hydrogen | 1 | 0 | 0 | 0 | 0 | 0 | 0 | 0 | 0 |
| h2o | EX_h2o(e) | Water | 10 | 1 | 1 | 1 | 1 | 1 | 1 | 1 | 1 |
| h2s | EX_h2s(e) | Hydrogen sulfide | 1 | 1 | 0 | 0 | 0 | 0 | 0 | 0 | 0 |
| hxan | EX_hxan(e) | Hypoxanthine | 1 | 1 | 0 | 0 | 0 | 0 | 0 | 1 | 0 |
| indole | EX_indole(e) | Indole | 1 | 0 | 0 | 0 | 0 | 0 | 0 | 0 | 1 |
| k | EX_k(e) | Potassium | 1 | 1 | 1 | 1 | 1 | 1 | 1 | 1 | 1 |
| lanost | EX_lanost(e) | lanosterol | 1 | 0 | 0 | 0 | 0 | 0 | 0 | 0 | 0 |
| meoh | EX_meoh(e) | Methanol | 10 | 0 | 0 | 0 | 0 | 0 | 0 | 0 | 0 |
| metsox_S_L | EX_metsox_S_L(e) | L-Methionine Sulfoxide | 1 | 1 | 1 | 1 | 1 | 1 | 1 | 1 | 1 |
| mg2 | EX_mg2(e) | Magnesium | 1 | 1 | 1 | 1 | 1 | 1 | 1 | 1 | 1 |
| mn2 | EX_mn2(e) | Mn2+ | 1 | 1 | 1 | 1 | 1 | 1 | 1 | 1 | 1 |
| mobd | EX_mobd(e) | Molybdate | 1 | 0 | 0 | 0 | 0 | 0 | 0 | 0 | 0 |
| mqn7 | EX_mqn7(e) | Menaquinone 7 | 1 | 1 | 0 | 0 | 0 | 0 | 0 | 0 | 0 |
| mqn8 | EX_mqn8(e) | Menaquinone 8 | 1 | 0 | 1 | 1 | 1 | 1 | 1 | 0 | 0 |
| na1 | EX_na1(e) | Sodium | 1 | 1 | 0 | 1 | 1 | 1 | 0 | 1 | 1 |
| nac | EX_nac(e) | Nicotinate | 1 | 1 | 1 | 1 | 1 | 1 | 1 | 1 | 1 |
| ncam | EX_ncam(e) | Nicotinamide | 1 | 0 | 0 | 0 | 0 | 0 | 0 | 0 | 0 |
| nmn | EX_nmn(e) | NMN | 1 | 0 | 1 | 1 | 1 | 0 | 1 | 0 | 1 |
| no2 | EX_no2(e) | Nitrite | 1 | 1 | 0 | 1 | 1 | 0 | 0 | 0 | 0 |
| no3 | EX_no3(e) | Nitrate | 1 | 0 | 0 | 0 | 0 | 0 | 0 | 0 | 0 |
| orn | EX_orn(e) | Ornithine | 1 | 0 | 1 | 1 | 1 | 1 | 1 | 1 | 0 |
| pheme | EX_pheme(e) | Protoheme | 1 | 1 | 1 | 1 | 1 | 1 | 1 | 0 | 0 |
| pi | EX_pi(e) | Hydrogenphosphate | 1 | 1 | 1 | 1 | 1 | 1 | 1 | 1 | 1 |
| pime | EX_pime(e) | Pimelate | 1 | 0 | 0 | 0 | 0 | 0 | 0 | 0 | 0 |
| pnto_R | EX_pnto_R(e) | (R)-Pantothenate | 1 | 0 | 1 | 1 | 1 | 1 | 1 | 1 | 1 |
| ptrc | EX_ptrc(e) | Putrescine | 1 | 0 | 1 | 0 | 1 | 1 | 1 | 0 | 1 |
| pydam | EX_pydam(e) | Pyridoxamine | 1 | 1 | 1 | 0 | 1 | 1 | 1 | 1 | 0 |
| pydx | EX_pydx(e) | Pyridoxal | 1 | 1 | 1 | 0 | 1 | 1 | 1 | 1 | 1 |
| pydx5p | EX_pydx5p(e) | Pyridoxal 5-phosphate | 1 | 0 | 0 | 0 | 0 | 0 | 0 | 0 | 0 |
| pydxn | EX_pydxn(e) | Pyridoxine | 1 | 1 | 1 | 0 | 1 | 1 | 1 | 1 | 0 |
| q8 | EX_q8(e) | Ubiquinone-8 | 1 | 0 | 1 | 1 | 1 | 1 | 1 | 0 | 0 |
| ribflv | EX_ribflv(e) | Riboflavin | 1 | 0 | 1 | 1 | 1 | 1 | 1 | 1 | 1 |
| sel | EX_sel(e) | Selenate | 1 | 0 | 0 | 0 | 0 | 0 | 0 | 0 | 0 |
| sheme | EX_sheme(e) | Siroheme | 1 | 1 | 1 | 1 | 1 | 1 | 1 | 0 | 0 |
| so4 | EX_so4(e) | Sulfate | 1 | 1 | 1 | 1 | 1 | 1 | 1 | 1 | 1 |
| spmd | EX_spmd(e) | Spermidine | 1 | 0 | 1 | 1 | 1 | 1 | 1 | 0 | 1 |
| thm | EX_thm(e) | Thiamin | 1 | 1 | 1 | 1 | 1 | 1 | 1 | 0 | 1 |
| thymd | EX_thymd(e) | Thymidine | 1 | 0 | 1 | 1 | 1 | 1 | 1 | 0 | 0 |
| ura | EX_ura(e) | Uracil | 1 | 1 | 1 | 1 | 1 | 1 | 1 | 1 | 1 |
| uri | EX_uri(e) | Uridine | 1 | 0 | 1 | 1 | 1 | 1 | 1 | 0 | 0 |
| xan | EX_xan(e) | Xanthine | 1 | 0 | 0 | 1 | 1 | 1 | 0 | 1 | 1 |
| zn2 | EX_zn2(e) | Zinc | 1 | 1 | 1 | 1 | 1 | 1 | 1 | 1 | 1 |
|  |  |  |  |  |  |  |  |  |  |  |  |
