# Supplementary material for: Quartet: Disentangling positive and negative components of microbial interactions
Source: PLoS Comput Biol. 2026 Jul 10;22(7):e1014502. doi: 10.1371/journal.pcbi.1014502 (PMC13384405; doi:10.1371/journal.pcbi.1014502)
Supplement: S9 Table — The cross-fed metabolites for all the species pairs of the oral microbial community we studied are listed. (DOCX) [file pcbi.1014502.s014.docx]

|  | **Smu** | **Smi** | **Sp** | **Sl** | **Ss** | **Bf** | **Lc** |
| --- | --- | --- | --- | --- | --- | --- | --- |
| **Av** | h, acald, asp_L, cys_L, for, gly, lys_L, nh4, phe_L, ppa, val_L, co2, csn, fru, glc_D, glyc, gua, lac_L, malt, met_L, nac, ser_L, thr_L | h, acald, ac, ala_L, asp_L, for, lys_L, succ, co2, dcyt, fru, gua, lac_L, met_L, nac, pi, ser_L | h, acald, ala_L, asp_L, for, h2o, lys_L, phe_L, ppa, succ, co2, dcyt, glu_L, glyc, gua, lac_L, met_L, nac, nh4, ser_L | h, acald, ac, ala_L, asp_L, cys_L, for, gly, lys_L, ppa, co2, dcyt, glc_D, glyc, lac_L, leu_L, malt, met_L, nh4, ser_L, thr_L, | h, acald, ac, ala_L, asp_L, for, lys_L, ppa, pro_L, arg_L, co2, csn, cys_L, glyc, gua, lac_L, met_L, nac, nh4, ser_L, thr_L | h, acald, ac, ala_L, asp_L, cys_L, for, h2o, lys_L, ser_L, succ, val_L, arg_L, co2, lac_L, nh4, phe_L | arg_L, co2, for, glu_L, ile_L, lac_L, leu_L, lys_L, nh4, phe_L, succ, val_L, h, asp_L, cys_L, lac_D, fru, gly, h2o, nac, thr_L |
| **Smu** |  | h2o, glc_D, csn, cytd, etoh, glu_L, gly, gua, lac_L, lys_L, met_L, ppa, h, acald, co2, dad_2, dgsn, gln_L, hista, ile_L, pi, ser_L, tym, uri, val_L | ade, h2o, csn, etoh, for, met_L, ppa, h, acald, asp_L, co2, dad_2, dcyt, glu_L, gua, hista, lac_L, lys_L, thr_L, uri | acald, h2o, glc_D, csn, gly, gua, lys_L, nac, succ, h, asp_L, co2, dgsn, hista, ile_L, lac_L, leu_L, ser_L, tym, uri | h, glc_D, co2, cytd, dad_2, fru, gln_L, lac_L, malt, met_L, thr_L, uri, acald, ade, h2o, asp_L, csn, cys_L, glu_L, lys_L, nac, ppa, tym | acald, ade, h2o, asp_L, for, met_L, nac, succ, ala_L, co2, cys_L, cytd, glu_L, lac_L, nh4, orn, phe_L, thr_L, val_L | acald, glc_D, co2, glu_L, glyc, lac_L, malt, met_L, nh4, h, ac, asp_L, cys_L, etoh, fru, gly |
| **Smi** |  |  | h, acald, and, ala_L, co2, dad_2, etoh, gln_L, gua, hista, leu_L, nac, pi, ser_L, succ, tym, uri, ade, asp_L, dcyt, glu_L, gly, h2o, lac_L, G6ppa | h, acald, co2, dad_2, fru, gln_L, gua, ile_L, lac_L, leu_L, lys_L, nac, pi, uri, ade, dcyt, dgsn, etoh, glu_L, h2o, hista, met_L, ppa, succ, tym | h, ala_L, co2, cytd, dad_2, fru, gln_L, gua, hista, leu_L, met_L, nac, pi, tym, uri, acald, ade, asp_L, csn, cys_L, dcyt, glu_L, h2o, ppa | h, acald, ac, ade, asp_L, for, gln_L, h2o, nac, pi, succ, ala_L, co2, cys_L, cytd, glu_L, gly, lac_L, orn, phe_L, val_L | acald, co2, for, lac_L, lys_L, met_L, nac, nh4, pi, succ, h, ala_L, asp_L, cys_L, etoh, gly |
| **Sp** |  |  |  | acald, ade, asp_L, dcyt, gly, gua, h2o, hista, lys_L, met_L, nac, ppa, pro_L, succ, tym, h, co2, dad_2, dgsn, glc_D, lac_L, malt, ser_L, thr_L, uri | h, asp_L, co2, cytd, dgsn, hista, ile_L, lac_L, leu_L, pro_L, tym, uri, acald, amp, arg_L, csn, cys_L, glu_L, gua, h2o, lys_L, met_L, nac, succ | h, acald, ade, asp_L, h2o, met_L, nac, pro_L, ser_L, succ, ala_L, co2, cys_L, cytd, orn, phe_L, thr_L | acald, co2, glu_L, glyc, lac_L, lys_L, met_L, nh4, ser_L, succ, h, ala_L, asp_L, cys_L, etoh, gly, h2o |
| **Sl** |  |  |  |  | h, ala_L, glc_D, co2, cytd, dgsn, gln_L, ile_L, malt, met_L, orn, ser_L, thr_L, uri, val_L, acald, h2o, asp_L, csn, cys_L, dcyt, gua, hista, lys_L, nac, nh4, ppa, succ, tym | h, ade, asp_L, etoh, ser_L, succ, acald, ala_L, co2, cytd, gly, lac_L, orn, phe_L | glc_D, co2, glu_L, lac_L, leu_L, lys_L, malt, met_L, nh4, succ, val_L, h, acald, ala_L, h2o, asp_L, cys_L, etoh, gly, nac |
| **Ss** |  |  |  |  |  | h, acald, ade, asp_L, h2o, lac_L, met_L, nac, val_L, ala_L, co2, cys_L, cytd, leu_L, phe_L, thr_L | acald, arg_L, co2, cys_L, glu_L, gly, glyc, ile_L, lac_L, leu_L, lys_L, met_L, nh4, pro_L, val_L, h, ac, ala_L, asp_L, etoh, h2o, nac, thr_L |
| **Bf** |  |  |  |  |  |  | acald, co2, cys_L, arg_L, glu_L, gly, ile_L, lac_L, leu_L, lys_L, nh4, phe_L, val_L, ac, h, ala_L, h2o, asp_L, etoh, for, nac, ser_L, succ |
