# Supplementary material for: Quartet: Disentangling positive and negative components of microbial interactions
Source: PLoS Comput Biol. 2026 Jul 10;22(7):e1014502. doi: 10.1371/journal.pcbi.1014502 (PMC13384405; doi:10.1371/journal.pcbi.1014502)
Supplement: Supplementary file 11 [file pcbi.1014502.s009.docx]

	Smu				Smi				Sp				Sl				Ss				Bf				Lc			
	[illegible]	[illegible]	[illegible]	[illegible]	[illegible]	[illegible]	[illegible]	[illegible]	[illegible]	[illegible]	[illegible]	[illegible]	[illegible]	[illegible]	[illegible]	[illegible]	[illegible]	[illegible]	[illegible]	[illegible]	[illegible]	[illegible]	[illegible]	[illegible]	[illegible]	[illegible]	[illegible]	[illegible]
**Av**	0.824	-0.458	0.822	-0.937	0.630	-0.411	0.551	-0.698	0.633	-0.458	0.549	-0.634	0.923	-0.522	0.959	-0.670	0.753	-0.443	0.725	-0.841	0.466	-0.522	0.649	-0.081	0.436	-0.522	0.321	-0.191
**Smu**					0.354	-0.738	0.247	-0.402	0.323	-0.738	0.221	-0.346	0.245	-0.681	0.190	-0.366	0.195	-0.596	0.182	-0.484	0.868	-0.937	0.513	-0.084	0.742	-0.937	0.605	-0.178
**Smi**									0.069	-0.698	0.085	-0.057	0.221	-0.466	0.209	-0.426	0.200	-0.423	0.235	-0.561	0.572	-0.698	0.583	-0.073	0.559	-0.698	0.537	-0.178
**Sp**													0.230	-0.422	0.235	-0.467	0.154	-0.365	0.203	-0.561	0.604	-0.634	0.327	-0.083	0.698	-0.634	0.714	-0.226
**Sl**																	0.276	-0.426	0.351	-0.663	0.890	-0.670	0.753	-0.089	0.318	-0.670	0.755	-0.226
**Ss**																					0.769	-0.841	0.406	-0.081	0.847	-0.841	0.800	-0.226
**Bf**																									0.275	-0.088	0.268	-0.226
