# Supplementary material for: Quartet: Disentangling positive and negative components of microbial interactions
Source: PLoS Comput Biol. 2026 Jul 10;22(7):e1014502. doi: 10.1371/journal.pcbi.1014502 (PMC13384405; doi:10.1371/journal.pcbi.1014502)
Supplement: Supplementary file 12 [file pcbi.1014502.s012.docx]

	Smu				Smi				Sp				Sl				Ss				Bf				Lc			
	[illegible]	[illegible]	[illegible]	[illegible]	[illegible]	[illegible]	[illegible]	[illegible]	[illegible]	[illegible]	[illegible]	[illegible]	[illegible]	[illegible]	[illegible]	[illegible]	[illegible]	[illegible]	[illegible]	[illegible]	[illegible]	[illegible]	[illegible]	[illegible]	[illegible]	[illegible]	[illegible]	[illegible]
**Av**	0.858	-0.470	0.844	-0.945	0.657	-0.421	0.563	-0.705	0.655	-0.430	0.522	-0.639	0.847	-0.046	0.590	-0.676	0.763	-0.446	0.720	-0.849	0.132	-0.526	1.183	-0.022	0.581	-0.526	1.440	-0.228
**Smu**					0.140	-0.006	0.004	-0.705	0.076	0.000	0.002	-0.639	0.033	0.000	0.002	-0.676	0.002	-0.945	0.208	0.000	0.818	-0.946	0.233	-0.078	0.796	-0.946	0.441	-0.192
**Smi**									0.015	-0.706	0.100	-0.014	0.018	-0.706	0.241	-0.018	0.020	-0.706	0.163	-0.024	0.558	-0.706	0.300	-0.071	0.640	-0.631	0.204	-0.228
**Sp**													0.232	0.000	0.002	-0.677	0.018	-0.640	0.057	0.000	0.615	-0.640	0.336	-0.086	0.730	-0.639	0.551	-0.228
**Sl**																	0.003	-0.676	0.220	0.000	0.655	-0.676	0.298	-0.080	0.768	-0.677	1.041	-0.228
**Ss**																					0.766	-0.849	0.403	-0.080	0.797	-0.849	1.878	-0.214
**Bf**																									0.658	-0.090	1.283	-0.228
